# Fine-scale characterization of the soybean rhizosphere microbiome via synthetic long reads and avidity sequencing

**DOI:** 10.1186/s40793-024-00590-5

**Published:** 2024-07-12

**Authors:** Brett Hale, Caitlin Watts, Matthew Conatser, Edward Brown, Asela J. Wijeratne

**Affiliations:** 1 AgriGro Incorporated, Doniphan, MO USA; 2 Arkansas Biosciences Institute, Arkansas State University, State University, AR USA; 3 College of Science and Mathematics, Arkansas State University, State University, AR USA; 4 College of Agriculture, Arkansas State University, State University, AR USA; 5 Present Address: Department of Animal Sciences, Purdue University, West Lafayette, IN USA

**Keywords:** Soybean, Rhizosphere, Microbiome, Soil biology, Amplicon sequencing, Synthetic long reads

## Abstract

**Background:**

The rhizosphere microbiome displays structural and functional dynamism driven by plant, microbial, and environmental factors. While such plasticity is a well-evidenced determinant of host health, individual and community-level microbial activity within the rhizosphere remain poorly understood, due in part to the insufficient taxonomic resolution achieved through traditional marker gene amplicon sequencing. This limitation necessitates more advanced approaches (e.g., long-read sequencing) to derive ecological inferences with practical application. To this end, the present study coupled synthetic long-read technology with avidity sequencing to investigate eukaryotic and prokaryotic microbiome dynamics within the soybean (*Glycine max*) rhizosphere under field conditions.

**Results:**

Synthetic long-read sequencing permitted de novo reconstruction of the entire 18S-ITS1-ITS2 region of the eukaryotic rRNA operon as well as all nine hypervariable regions of the 16S rRNA gene. All full-length, mapped eukaryotic amplicon sequence variants displayed genus-level classification, and 44.77% achieved species-level classification. The resultant eukaryotic microbiome encompassed five kingdoms (19 genera) of protists in addition to fungi – a depth unattainable with conventional short-read methods. In the prokaryotic fraction, every full-length, mapped amplicon sequence variant was resolved at the species level, and 23.13% at the strain level. Thirteen species of *Bradyrhizobium* were thereby distinguished in the prokaryotic microbiome, with strain-level identification of the two *Bradyrhizobium* species most reported to nodulate soybean. Moreover, the applied methodology delineated structural and compositional dynamism in response to experimental parameters (i.e., growth stage, cultivar, and biostimulant application), unveiled a saprotroph-rich core microbiome, provided empirical evidence for host selection of mutualistic taxa, and identified key microbial co-occurrence network members likely associated with edaphic and agronomic properties.

**Conclusions:**

This study is the first to combine synthetic long-read technology and avidity sequencing to profile both eukaryotic and prokaryotic fractions of a plant-associated microbiome. Findings herein provide an unparalleled taxonomic resolution of the soybean rhizosphere microbiota and represent significant biological and technological advancements in crop microbiome research.

**Supplementary Information:**

The online version contains supplementary material available at 10.1186/s40793-024-00590-5.

## Background

Microbial symbionts demonstrate the propensity to overcome basal plant immunity, after which time they may occupy host compartments and therewith engage in mutualism, commensalism, and/or parasitism [1]. These interrelationships exist across a context-dependent, temporally plastic continuum [2, 3] and are pivotal for plant health and physiology [4]. This is exemplified in the rhizosphere, defined as the soil region directly influenced by root exudates, where selective pressures imposed by a host encourage the colonization of fitness-promoting microorganisms [5]. Consequently, the assembly, function, and sustenance of the rhizosphere microbiome have become focal points of intensive research endeavors, particularly within food crop systems, due to their intrinsic link to plant health and broader implications for agricultural sustainability [6].

The Fabaceae (Leguminosae) serve as model systems for rhizosphere microbiome research given their capacity to recruit diazotrophic bacteria for atmospheric nitrogen fixation [7]. This is well evidenced by soybean (*Glycine max*), a global staple crop for which rhizosphere microbiome dynamics have been extensively studied. Mendes et al. [8] found that bacterial assemblages in the soybean rhizosphere were less diverse than in corresponding bulk soils, reflecting preferential selection of microbiota adept in N, Fe, P, and K metabolism. Likewise, Zhang et al. [9] surveyed 51 soybean fields across China, revealing that while soil pH predominantly influenced bacterial communities, eukaryotic assemblages were more responsive to Mg levels. Biotic stressors including *Fusarium virguliforme* [10], *Phytophthora sojae* [11], and *Heterodera glycines* [12] have also been implicated to modulate soybean rhizosphere microbiome structure, as have tillage [13–15], biological product/fertilizer application [16], host growth stage [13, 17], host genotype [18], and other edaphic parameters [14, 15]. Nonetheless, one must exercise caution when interpreting such findings, especially when bridging taxonomy with function, given the incomplete representation of soil microbiota in public databases as well as the inherent constraints of common microbiome profiling methodologies (reviewed at-length by Baldrian [19]).

Amplicon sequencing is a primary method for microbiome profiling, as microorganism identification is not restricted by culturing capacity [20] and workflows are resource-efficient in comparison to shotgun metagenomic approaches [21]. Leveraging PCR amplification of marker genes, amplicon sequencing entails primer annealing to conserved regions within rRNA operons and the use of adjacent hypervariable regions for taxonomic classification [22]. In prokaryotes, the 16S rRNA gene is targeted due to its nine hypervariable regions (V1 to V9) interspersed with highly conserved sequences [23]. For eukaryotes, multiple regions within the rRNA operon can be utilized. The 18S rRNA gene (SSU or Small Subunit) serves a similar purpose to the 16S in prokaryotes, providing broad taxonomic identification [24]. For heightened resolution, the Internal Transcribed Spacer (ITS) regions ITS1 and ITS2 are chosen [25]. These regions are located between the SSU and the 5.8S rRNA genes, and between the latter and the Large Subunit (LSU or 28S rRNA gene) in the operon [26]. PCR amplicon sequencing of such regions produces amplicon sequence variant (ASV) read numbers that estimate organism abundances with reasonable precision, though estimate accuracy can vary significantly. Despite improved coverage of marker gene-based approaches [27], traditional short-read sequencing technologies are limited to the interrogation of few hypervariable regions, rendering region-specific bias [28], uncertain/erroneous taxonomic classification [29], and limited classification beyond genus level [19–21]. Many in the field recommend using long read-based sequencing strategies to overcome such limitations (i.e., Oxford Nanopore Technology [ONT] and Pacific Biosciences [PacBio]) [30, 31]; yet, ONT has demonstrated inferior accuracy compared to other sequencing platforms [32] and PacBio remains relatively cost-prohibitive [31]. To derive tractable biological inference from microbiome profiling, it is imperative to employ methodologies with enhanced resolution, accuracy, and accessibility.

The LoopSeq platform by Element Biosciences (formerly Loop Genomics) is a synthetic long-read (SLR) sequencing method that addresses many of the defined challenges surrounding amplicon-based microbiome profiling. To this end, each parent DNA molecule in a sample is barcoded with a unique molecular identifier (UMI) which is thereafter distributed intramolecularly across the molecule [33]. Post-fragmentation and sequencing, short reads sharing a UMI are assembled de novo to reconstruct the entire parent molecule sequence. This approach employs a consensus-driven error correction system that renders a higher fraction of error-free reads compared to PacBio circular consensus reads and the cited ONT per-base error rate [33]. LoopSeq additionally minimizes the formation of PCR amplicon chimeras, as chimeric molecules are unlikely to contribute to the consensus unless they dominate the reads for a given UMI [33]. In practice, LoopSeq outperformed V3-V4 short-read sequencing in terms of taxonomic resolution and identification accuracy for human gut microbiota [34], and was superior to V4 and PacBio in accuracy and cost for soybean rhizosphere microbiome profiling (~ 90% per-Mb cost reduction compared to PacBio) [29]. Thus, LoopSeq SLR technology holds transformative potential for comprehensive and precise microbiome analysis across diverse study systems.

**Fig. 1:**
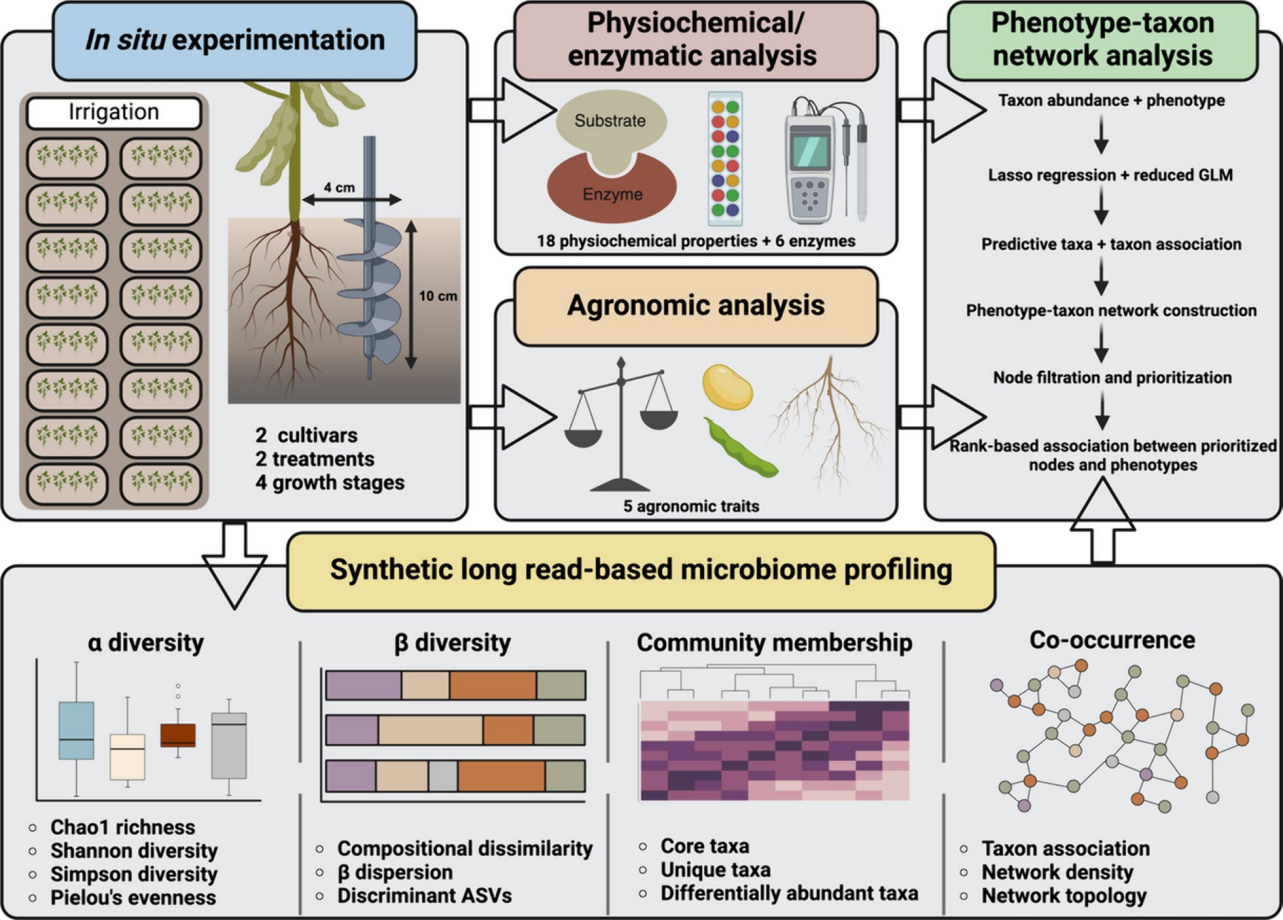
Schematic representation of the experimental design. This graphic was created using BioRender (Biorender.com)

In the present study, the LoopSeq SLR platform was used to profile the soybean rhizosphere microbiome under field conditions. The experimental design incorporated two commercially available soybean cultivars with contrasting levels of tolerance to *F. virguliforme*-induced Sudden Death Syndrome (SDS), the absence/presence of an in-furrow/foliar biostimulant regimen, and four growth stages spanning vegetative and reproductive development (Fig. 1). Following DNA isolation and library preparation, the UMI-tagged fragments were sequenced using avidity chemistry, which independently optimizes DNA template traversal and nucleotide identification, achieving an accuracy surpassing one error per 10,000 bp [35]. The resulting short reads were assembled into SLRs spanning all nine hypervariable regions for the 16S rRNA gene or the entire 18S-ITS1-ITS2 region of the eukaryotic rRNA operon. Complementary to standard microbiome assessment, 24 edaphic properties and five agronomic traits were measured, integrated into phenotype-taxon networks, and used to prioritize taxa with putative ecological relevance (Fig. 1).

## Materials and methods

### Site description and management

The test site was located at the Arkansas State University Agricultural Teaching and Research Center (35° 50′ 16″ N, 90° 40′ 00″ W). The cropland consisted of a Collins silt loam (Coarse-silty, mixed, active, acid, thermic Aquic Udifluvent) with a 0 to 1% slope and had been used historically for the cultivation of a corn (*Zea mays*)-soybean rotation in the absence of tillage. A cover crop blend of black oat (*Avena strigosa*), Austrian winter pea (*Pisum sativum* L. ssp. *sativum* var. *arvense*), and buckwheat (*Fagopyrum esculentum*) was sown during the fallow period using a Great Plains End Wheel No-Till Compact Drill (Great Plains Manufacturing Incorporated, Salina, KS, USA) with 19.05-cm row spacing. In addition, cattle (*Bos taurus*) were grazed on the site for 10 d.

At the beginning of the growing season, the cover crop was terminated using 1.46 L ha^−1^ Roundup PowerMAX 3 (Bayer CropScience, Monheim, Germany) plus 0.37 L ha^−1^ Verdict (BASF, Ludwigshafen, Germany). Seven d after, the test site was fertilized with P_2_O_5_ (51.45 kg ha^−1^), K_2_O (99.87 kg ha^−1^), and S (3.03 kg ha^−1^). Four passes were made with a John Deere tandem disc (Deere and Company, Moline, IL, USA), and were followed by one pass with a Triple K field cultivator (Kongskilde Agriculture, Albertslund, Denmark). Raised seedbeds were then formed using a 2-row Hipper Roller (Brandt, Springfield, IL, USA) with 76.2-cm row spacing. Approximately 2.34 L ha^−1^ Command® 3ME (FMC Corporation, Philadelphia, PA, USA) plus 0.37 L ha^−1^ Verdict® were applied for pre-emergent weed control 2 d prior to the soybean planting date. Soybeans (cultivars described below) were then planted with a total row length of 106.68 m at a seeding rate of 345,947 seeds ha^−1^ using a John Deere 1705 4-row vacuum planter (Deere and Company). A second fertilizer application was made 1 d after planting and was equivalent to the first. Following plant emergence, 3.51 L ha^−1^ Warrant (Bayer CropScience) and 2.34 L ha^−1^ Roundup PowerMAX 3 were applied for the management of yellow nutsedge (*Cyperus esculentus* L.). Manual weed removal was performed weekly throughout the growing season. Moreover, a channeled (furrowed) surface irrigation system ran along the north side of the test site and was used to deliver one acre-inch water (~ 102,789 L) to the crop. Irrigation began the first week in July and was performed weekly until the third week in September. Weather data for the growing season were obtained from Visual Crossing Corporation (https://www.visualcrossing.com/) and can be found in Additional file 1.

### Experimental design

A randomized split-block design comprising eight rows of BASF Credenz soybeans was used in this study. Four rows consisted of the cultivar CZ4979X (maturity group 4.9; SDS-tolerant), while the other four were CZ4810X (maturity group 4.8; SDS-susceptible). Both 4-row sections were divided into 6.096-m plots separated by 7.62-m buffer zones, yielding 8 randomized plots (4 control and 4 treatment) for each section (16 total). At the early vegetative stage (V1 – one set of unfolded trifoliate leaves), the biostimulant IgniteS^2^ (AgriGro Incorporated, Doniphan, MO, USA) was applied to the base of plants in treatment plots at a rate of 1.17 L ha^−1^. The biostimulant FoliarBlend (AgriGro Incorporated) was applied to the foliage at mid-vegetative (V3 – third set of unfolded trifoliate leaves) and early reproductive (R3 – full flower inflorescence/reproductive stage) stages at a rate of 1.17 L ha^−1^. Biostimulant applications were made with a backpack sprayer within the inner 2 rows for each treatment plot. Soybean growth stages were defined by Fehr and Caviness [36].

### Soil sampling and DNA isolation

From each plot, a composite sample comprising 10 soil cores was taken at the following growth stages: early vegetative (V1—first unfolded trifoliate, preceding biostimulant application), late vegetative (V6—6th node, preceding anthesis), early reproductive (R2—full flower inflorescence/reproductive stage), and late reproductive (R6—full pod development) (i.e., 640 cores reduced to 64 composite samples). Spatially distributed selective pressures reduce microbial diversity in proximity to the root surface [37, 38], which must be considered during sampling. Therefore, cores were collected with a 2 cm-diameter auger 4 cm from the base of the plant to a depth of 10 cm (Fig. 1). Composite samples were collected in treatment- and cultivar-specific vessels to minimize cross-contamination. In addition, augers were sterilized in a 20% bleach solution (7.4% NaClO) for 5 min and rinsed thoroughly with water between plot samplings. Collection and handling procedures were consistent across all samples. Following collection, each composite sample was homogenized, sieved to 2 mm, and subdivided for downstream physiochemical, enzyme, and microbiome analyses. Subsamples for enzyme and microbiome analyses were placed immediately into sterile 50 mL conical tubes, freeze-dried on solid CO_2_, and stored at − 80 °C.

DNA was isolated from 250 mg of each sample using the DNeasy PowerLyzer PowerSoil Kit (Cat #12855-100) according to the manufacturer’s protocol (QIAGEN, Hilden, Germany). Following the addition of soil, PowerBead Solution, and Solution C1 to the PowerBead tube, cells were lysed with a Precellys 24 tissue homogenizer with the following program: 3000 RPM × 30 s duration x three cycles with a 20 s delay between cycles. A Qubit fluorometer (Thermo Fisher Scientific, Waltham, MA, USA) paired with a dsDNA high-sensitivity assay kit (Cat #Q32851) was used subsequently to estimate DNA concentration. DNA purity was approximated from 260/230 and 260/280 nm absorbance ratios using a NanoDrop ND-1000 spectrophotometer. A sample without soil served as a ‘kitome’ control during DNA isolation [39]. Additional positive (ZymoBIOMICS™ Microbial Community DNA Standard [Cat #D6305]) and negative (template-free sample) controls were included for barcoding and sequencing steps.

### Amplicon sequencing and SLR assembly

Sequencing libraries were prepared at Element Biosciences (San Diego, CA, USA) using the Amplicon LoopSeq for AVITI (Cat #840–00002), Extension LoopSeq for AVITI (Cat #840–00003), and Element Elevate™ Library Circularization Kit (Cat #830–00001) as per manufacturer guidelines. Initial steps involved target enrichment and PCR amplification of the 16S and 18S-ITS rRNA regions using the following primers: 16S Fwd (5′-AGAGTTTGATCMTGGCTCAG-3′), 16S Rev (5′-TACCTTGTTACGACTT-3′), 18S-ITS Fwd (5′-TACCTGGTTGATYCTGCCAGT-3′), and 18S-ITS Rev (5′-GGTTGGTTTCTTTTCCT-3′, 5′-TAAATTACAACTCGGAC-3′, 5′-TCCTCCGCTTWTTGWTWTGC-3′, 5′-CTBTTVCCKCTTCACTCG-3′). Subsequently, UMIs and a LoopSeq index were integrated into each sample, and the barcoded samples were calibrated, amplified, and multiplexed. Library preparation was then performed by distributing each UMI to a random position within the respective parent molecule, fragmenting the barcoded molecules at each UMI position, and adding Element indexes and adapters. The final library was circularized and sequenced on the Element AVITI System (Cat #880-00001). All amplification conditions are provided at https://github.com/brett-hale/Hale_2024_SLR.git. The Bases2Fastq software (v1.4.0) was used to convert the bases files into FASTQ files and de-multiplex the pooled library based on index sequences.

Data processing was performed with the Element Biosciences cloud-based platform, largely following the bioinformatics pipeline established by Callahan et al. [33]. First, adapter sequences were removed from short reads using Trimmomatic (v0.36) [40], and trimmed reads were de-multiplexed based on their LoopSeq Index. Reads within a grouped sample were then binned by UMI and further processed through SPAdes (v3.9) [41], allowing the de novo assembly of SLRs spanning the full length of the defined rRNA operon sequence. The resultant SLRs were further processed and thereafter clustered into ASV bins of 100% sequence homology with the *DADA2* R package (v1.28.0) [42] employing the specifications outlined by Callahan et al. [33]. Taxonomic assignments were conducted using BLAST, with a criterion of 97% sequence similarity. Prokaryotic classifications were based on the SILVA SSU database (v138) [43], while eukaryotic identifications used the UNITE database (all eukaryotes v8) [44, 45]. Short-read and SLR summary data are provided in Supplementary Fig. 1, Additional file 2; Additional file 3; and Additional file 4.

### Microbiome statistical analysis

The ASV count matrices, taxonomic assignments, and sample metadata were imported into RStudio (v4.2.2) [46] and combined to create ‘phyloseq’ objects with the *phyloseq* package (v1.42.0) [47]. ASVs mapped to kingdoms ‘Viridiplantae’ and ‘Metazoa’ were removed subsequently from the 18S-ITS object to accentuate true eukaryotic microbiota. Prior to statistical analysis, samples were decontaminated based on ASVs present in the two negative controls using the ‘isContaminant’ function in the *decontam* package (v1.13) with a 0.5 prevalence probability threshold [48]. Within-sample (α) diversity was then investigated by estimating the Chao1 index [49], Simpson diversity [50], and Shannon diversity [51] using the ‘estimate_richness’ function in *phyloseq*. Pielou's evenness [52] was assessed with the ‘evenness’ function in the *microbiome* package (v1.2.1) [53]. Rank-based measures of association between the eukaryotic and prokaryotic Chao1 indices, Shannon diversity, and between the Chao1 index and Shannon diversity were inferred using the 'cor.test' function in the R package *stats* (v4.2.2), leveraging Spearman’s *ρ* statistic [54]. The package *ggplot2* (v3.4.2) [55] was used for data visualization.

The 16S and 18S-ITS α diversity datasets were divided into the baseline measurement (V1) and growth stages succeeding biostimulant application (V6, R2, and R6). Within each partitioned dataset, the assumption of normality was assessed for Shannon diversity and Chao1 using the Shapiro–Wilk test [56] in the *stats* package. In instances when the normality assumption was not met (*p* value < 0.05), data were fitted to five probability distributions using the ‘fitdist’ function in the *fitdistrplus* package (v1.1–11) [57], and the best-fitting distribution was inferred from the minimum Akaike information criterion (AIC). Distribution fit was supported qualitatively with quantile–quantile plots generated with packages *car* (v3.0–12) [58] and *MASS* (v7.3–54) [59]. Generalized linear mixed models (GLMMs) were then implemented with package *glmmTMB* (v1.1.7) [60]. Treatment, cultivar, growth stage, and first-, second-, and third-order interactions were incorporated as explanatory variables, with field location incorporated as a random effect. Automated model selection was performed with the *MuMin* (v1.47.5) [61] ‘dredge’ function, with selection constrained to models containing treatment, cultivar, and growth stage. The best-fitting model was selected by minimum AICc (second-order AIC) as recommended by Burnham and Anderson [62]. Model fit was assessed with standardized and deviance residuals leveraging package *stats*. Additionally, 250 datasets were simulated and used to calculate an empirical cumulative density function, the residuals of which were examined through quantile–quantile and residual-fitted value plots. Binomial and Poisson models were checked explicitly for overdispersion using ‘check_overdispersion’ from the *performance* package (v0.10.3) [63]. Moreover, fixed effects retained in final models were further evaluated by hierarchical partitioning of marginal R^2^ values using *glmm.hp* (v0.1–0) [64] and through power analyses implemented with the ‘powerSim’ function of the *SIMR* package (v1.0.6) [65]. Marginalized coefficients were extracted for models applying a nonlinear link function with the ‘marginal_effects’ function of *margins* (v0.3.26) [66], and the mean coefficients (denoted hereafter as “mean estimates”, or “ME”) were visualized with *ggplot2*.

Following α diversity estimation, unmapped ASVs were removed from the phyloseq objects, and count matrices were normalized by cumulative sum scaling (CSS) with *metagenomeSeq* (v1.40.0) [67]. Condition-specific (treatment-cultivar-growth stage) compositions were then visualized at the phylum level with *ggplot2*. Thereafter, compositional dissimilarity was assessed at ASV level for samples preceding and succeeding biostimulant application using Bray–Curtis, Euclidean, and Jaccard distances [68, 69] with the function ‘distance’ in *phyloseq*. Statistical trends in community structure were inferred by conducting permutational multivariate analysis of variance (PERMANOVA) with the defined dissimilarity matrices. Each PERMANOVA was performed independently with the ‘adonis2’ function in *vegan* (v2.6–4) [70] specifying treatment, cultivar, and growth stage as well as first- and second-order interactions as explanatory variables, and with 9,999 permutations constrained by field location. Compositional variance attributed to each explanatory variable was inferred from PERMANOVA R^2^ values. Multivariate homogeneity of group dispersions was then analyzed with the *vegan* function ‘betadisper’. Analysis of variance (ANOVA) was used subsequently to determine if distances to group centroids varied significantly between fixed effect levels. PERMANOVA and dispersion test results were consistent across dissimilarity matrices (Supplementary Table 1, 2, Additional file 2); therefore, representative ordinations of Bray–Curtis dissimilarity were plotted using Principal Correspondence Analysis (PCoA) and Non-metric Multidimensional Scaling (NMDS) with the ‘ordinate’ *phyloseq* function and *ggplot2*. In the post-biostimulant application datasets, the ‘simper’ function from the *vegan* package was employed to determine the contribution of individual ASVs to the Bray–Curtis dissimilarity between levels of each fixed effect [71]. The top five ASVs for each pairwise comparison were identified, their taxonomy extracted from the phyloseq object, and their contribution percentage visualized using the *ComplexHeatmap* package (v2.14.0) [72]. Lastly, Bray–Curtis dissimilarity matrices were reconstructed with CSS-normalized counts from both mapped and unmapped full-length ASVs, compositional trends were assessed via PERMANOVA and β dispersion estimation, and parallels were discerned between the full and partial dataset analyses.

The taxonomic resolution achieved with both 16S and 18S-ITS amplicon sequencing enabled the retrieval of functional profiles for most mapped ASVs. Genus-level eukaryotic functions were sourced from the FungalTraits database (v1.2) [73]. Prokaryotic functional profiles were derived at the species level from Bac*Dive*, a bacterial/archaeal metadatabase maintained by the German Collection of Microorganisms and Cell Cultures [74]. The ‘retrieve’ function of the R package *BacDive* (v0.8.0) [75] was employed with parameters set as “query = species list” and “search = taxon”. The resulting metadata was narrowed to terms including “aerobe”, “philic”, “path”, and “gram”, reflecting O_2_ tolerance, temperature range, pathogenicity, and gram stain, respectively. The collated eukaryotic and prokaryotic data were then combined into a comprehensive functional data frame capturing Structure (eukaryotic fruiting body/prokaryotic gram stain), Growth (eukaryotic growth form/prokaryotic temperature range), Environment (eukaryotic aquatic habitat/prokaryotic O_2_ tolerance), and Lifestyle/Pathogenicity. This data informed and contextualized taxa highlighted in subsequent analyses.

Community membership was determined at genus and species levels for eukaryotic and prokaryotic communities, respectively, which were the lowest taxonomic classifications to which all full-length, mapped ASVs could be identified. Core taxon analysis was performed with the *microbiome* package by first converting CSS-normalized counts to relative abundances with the function ‘transform’ and specification ‘compositional’, and by subsequently obtaining taxa with a prevalence ≥ 0.5 with the ‘core_members’ function. The relative abundance of core taxa was visualized with the *ComplexHeatmap* package. The ASVs unique to a fixed effect level were retrieved using the ‘unique_taxa’ function of the *phylosmith* package (v1.0.6) [76]. The full dataset was used to identify unique ASVs between cultivars and growth stages. The analysis was repeated for treatment with datasets partitioned into the baseline measurement and samplings succeeding biostimulant application, and ASVs retrieved exclusively from the latter dataset were deemed unique to a level of treatment. This information was used to identify unique and shared taxa across fixed effects and domains, which were visualized with Venn diagrams constructed with *ggvenn* (v0.1.10) [77] as well as with *ComplexHeatmap*.

Differentially abundant taxa were identified between fixed effect levels with *MaAsLin2* (Microbiome Multivariable Associations with Linear Models) (v1.12.0) [78]. CSS-normalized counts for taxa with a minimum prevalence > 0.1 were fitted with a zero-inflated negative binomial (ZINB) regression model composed of treatment, cultivar, and growth stage as fixed effects and field location as a random effect. *Maaslin2* inherently corrects for multiple testing using the Benjamini–Hochberg approach, thus taxa with a *q*-value < 0.25 were deemed significant as recommended by the package authors [78] and as reported in the literature [79, 80]. Consistent with unique taxon identification, the full dataset was used to identify differentially abundant taxa between levels of cultivar and growth stage, with partitioned datasets deployed for treatment. Regarding the latter, taxa exhibiting statistically significant, consistent directional changes (both positive or negative coefficients) across the baseline and post-treatment datasets were excluded. Conversely, taxa that displayed opposing directional changes between the two datasets (i.e., positive in one and negative in the other), or were present exclusively in the post-treatment dataset, were retained. Log-normalized False Discovery Rate (FDR; − sign[coefficient)*log(*q*-value]) for differentially abundant taxa were visualized with *ComplexHeatmap*.

To further assess community membership, eukaryotic and prokaryotic *phyloseq* objects were combined and conglomerated at the genus level with the ‘merge_phyloseq’ and ‘conglomerate_taxa’ functions, respectively, of the *phylosmith* package. A global pairwise Spearman co-occurrence network was then constructed by obtaining significant positive and negative associations (*ρ* >  ± 0.6, *p* value < 0.05) with the *phylosmith* ‘co_occurrence’ function. The *p*-values were corrected for multiple testing using the *stats* function ‘p.adjust’ specifying FDR correction. Associations with a *q*-value < 0.05 were visualized with the *phylosmith* function ‘co_occurrence_network’ with nodes representing genera and edges representing positive and negative associations.

Condition-specific co-occurrence networks (*n* = 16) were constructed as described for the global networks, and the *phylosmith* ‘network_layout_ps’ function was used subsequently to create a graph object from each set of co-occurrences. Comparisons of network topology were then performed by calculating centralization degree (the concentration of network centrality), cluster count, connectance (the proportion of possible connections that are present), edge count, node count, and mean degree (the average degree [number of connections] of nodes) with the ‘net_properties’ function in the *ggClusterNet* R package (v0.1.0) [81]. Giant component size (the size of the largest connected component) and modularity (the extent to which a network can be divided into non-overlapping communities) were determined with the *igraph* R package (v1.4.2) [82]. For the latter metric, nodes were assigned to communities using the Walktrap algorithm [83] implemented with the ‘cluster_walktrap’ function, and modularity was calculated with the ‘modularity’ function using the community membership vector as input. Moreover, microbial co-occurrence networks inherently exhibit a scale-free topology in which node degrees follow a power-law distribution [84]. To this end, condition-specific graph objects were converted to degree distribution vectors with the *igraph* ‘degree_distribution’ function, and a power-law distribution was fitted to each vector with the ‘fit_power_law’ *igraph* function. The Kolmogorov–Smirnov (KS) test statistic was used to quantify the distance between node degree distribution and a power-law distribution. Nodes were prioritized for each network by assigning Kleinberg’s hub centrality scores [85] with the *igraph* function ‘hub_score’ with logical scaling applied. Nodes with a hub score > 0.2 were considered hubs, which was consistent with prior studies [86]. To further support predicted co-occurrences, global and condition-specific networks were reconstructed with Pearson associations [87] following the described methodology. Unique and shared co-occurrences and nodes between the association methods were visualized with *ggvenn* Venn diagrams. Additional rank-based Spearman associations were inferred between edge count and node count (network properties used commonly to reflect density) and the remaining topological features for each set of condition-specific networks. Furthermore, Friedman rank sum tests [88] were applied with the *stats* package to assess the differential ranking of conditions between association methods for each topological feature. Network membership was visualized using *ComplexHeatmap*.

Significant microbial co-occurrences, identified through global networks of one or both association methods, were combined with edaphic and agronomic parameters (*n* = 24 and 5, respectively; methodology described hereafter) to construct phenotype-taxon networks using the *PhONA* (phenotype-OTU network analysis) R package (v0.2) [89]. This package first employs lasso regression to identify taxa predictive of a defined phenotype. From these predictive taxa, *PhONA* constructs a generalized linear model (GLM) and subsequently integrates the GLM with the user-provided co-occurrence matrix [89]. All sample information was used for edaphic parameter network analysis, while agronomic parameter network analysis was performed with samples from the R6 growth stage. Resulting phenotype-taxon networks were reconstructed with *igraph*, and network membership was visualized using *ComplexHeatmap*.

The *PhONA* package assigns modularity roles to each node by computing connectivity (within-module z-score of edge weights) and a participation coefficient (distribution of a node's links across different modules), methodology consistent with the *rnetcarto* R package [89, 90]. In this study, the modularity analysis was augmented with Kleinberg's hub centrality (computed as described for co-occurrence networks), providing a more nuanced differentiation of core network nodes based on information dissemination roles. The three metrics were individually normalized to a [0,1] scale and then combined with equal weights to compute a composite centrality score for each node in the phenotype-taxon network. Nodes were prioritized by first filtering to those with a ≥ 0.2 prevalence across all samples in an effort to minimize biased associations while retaining putative specialization [91]. Thereafter, genera were ranked by mean composite score, accentuating those for which a phenotype could indicate a niche specialization in addition to those central across all networks. The top 20 genera were obtained for each parameter type (edaphic and agronomic), and rank-based measures of association were inferred between genera using CSS-normalized counts, as well as between genera and edaphic/agronomic parameters.

### Edaphic parameter estimation

Freeze-dried subsamples were sent to Ward Laboratories Inc. (Kearney, NE, USA) for quantitative assessment of β-glucosidase (GB3), N-acetyl-β-glucosaminidase (NAG), phosphodiesterase (PDE), alkaline phosphatase (ALP), acid phosphatase (ACP), and arylsulfatase (ARS). The GB3 assays were based on Moscatelli et al. [92] methods, while NAG assays employed procedures from Deng and Popova [93] and Parham and Deng [94]. Both phosphatase enzymes were analyzed using Nannipieri et al. [95] protocols. The ARS assays followed the methodologies of Tabatabai and Bremner [96] and Klose et al. [97]. Each assay utilized 2 g of soil, and enzymatic activities were quantified using a BioTek Epoch 2 Microplate Spectrophotometer (Agilent Technologies, Santa Clara, CA, USA).

The remaining subsamples were kept in plastic bags at room temperature until shipment to Waypoint Analytical (Memphis, TN, USA) where the following macro- and micronutrient levels were measured following standard Mehlich 3 Extraction procedure [98]: B, Ca, Cu, Fe, K, Mg, Mn, Na, P, S, and Zn. Additionally, soil pH was assessed using the 1:1 soil–water ratio method and buffer pH using the Shoemaker–McLean–Pratt (SMP) procedure [99]. Soil organic matter (SOM) was estimated using the Loss-on-ignition method [100] and N in the form NO_3_- as defined by Swift and Sparks [101]. Lastly, the percent saturation of Ca, H, K, Mg, and Na were used to estimate cation exchange capacity (CEC) in milliequivalents per 100 g (meq/100 g) of soil. Detailed protocols for all edaphic measurements can be found in Gavlak et al. [102].

Pairwise rank-based measures of association were inferred across all parameters. Furthermore, differences between fixed effect levels for each parameter were assessed using GLMMs as described previously.

### Agronomic parameter estimation

When soybean plants reached physiological maturity (R8—95% of pods have reached their full mature color), three plants per plot (*n* = 48) were selected randomly for measurement of the following characteristics: pods per plant (exclusive to those containing ≥ 1 full seed), root biomass, and aboveground biomass. For biomass measurements, roots were rinsed gently with tap water to remove substrate and plants oven-dried at 60 °C for 72 h. Roots were then cut at the soil line (~ 4 cm above the 1st lateral root) and root and aboveground dry weight determined independently. Plant selection and data collection were performed by researchers blinded to experimental conditions.

One hundred-seed weight and theoretical grain yield were also determined at the R8 growth stage. First, an area of 1.16 m^2^ was selected randomly from each plot and manually harvested. Collected plants were threshed using a stationary Plot Master Combine (ALMACO, Nevada, IA, USA). Seed moisture was then assessed using a mini GAC® Plus Grain Moisture Tester (Dickey-John Corporation, Auburn, IL, USA) and seed weight calculated at a 13% moisture base. Theoretical grain yield was determined in kg ha^−1^ with the corrected seed weight.

GLMMs were used to discern differences between levels of treatment, cultivar, and treatment-cultivar interactions. In instances where multiple plants were selected per plot, plant replicates were modeled as nested random effects to account for a lack of independence among observations.

### Additional information

Plant lodging was observed around R5 (beginning of seed fill). In addition, Cercospora leaf blight (*Cercospora kukuchii*) and soybean stem borer (*Dectes texanus*) damage occurred between R6 and R8 (full seed to maturity).

## Results

### SLRs enabled a taxonomically resolved assessment of the soybean rhizosphere microbiome

The present study employed SLR technology in tandem with avidity sequencing to explore the composition and structure of the soybean rhizosphere microbiome. For 18S-ITS amplicon sequencing targeting eukaryotes, this method generated a collective 852 M short reads assembled into 1.4 M SLRs averaging a length of 1108.31 ± 663.99 bp (full and partial length) (Supplementary Fig. 1, Additional file 2; Additional file 3). Similarly, the full-length SLRs were 1084.69 ± 671.98 bp. From these, 1,014 denoised ASVs were classified at the genus level using the Ensemble reference database, and 44.77% could be further classified at the species level. The mapped eukaryotic SLRs were 2328.54 ± 213.7 bp. Conversely, the 16S (prokaryotic) dataset comprised 192 M short reads, resulting in 1.4 M SLRs with a mean length of 1362.44 ± 291.46 bp (Supplementary Fig. 1, Additional file 2; Additional file 4). The full-length contigs were 1,476.06 ± 70.51 bp in length. Mapping these to the Silva138 database permitted the identification of 895 ASVs, all classified at the species level, with 207 (23.13%) achieving strain-level classification. The mapped prokaryotic SLR length was consistent with that of all full-length SLRs, being 1477.64 ± 25.15 bp.

The soybean rhizosphere microbiome displayed a diverse taxonomic profile spanning seven kingdoms. The eukaryotic fraction consisted primarily of partly aquatic, saprotrophic fungi that exhibit perithecial fruiting bodies and filamentous mycelial growth forms, while the prokaryotic fraction largely comprised gram-negative, mesophilic, aerobic bacteria. Beyond bacteria and fungi, protist populations from five kingdoms were observed: Alveolata, characterized by membrane-bound sacs (alveoli) beneath the plasma membrane [103]; Apusozoa, flagellated unicellular eukaryotes [104]; Heterolobosa (i.e., Heterolobosea), protists with both amoeboid and flagellated stages [105]; Rhizaria, identified by their thread-like pseudopodia [106]; and Stramenopila, which encompasses diatoms, brown algae, and oomycetes, differentiated by their heterokont flagella [107].

### Growth stage and spatial heterogeneity best explained microbiome structure

**Fig. 2:**
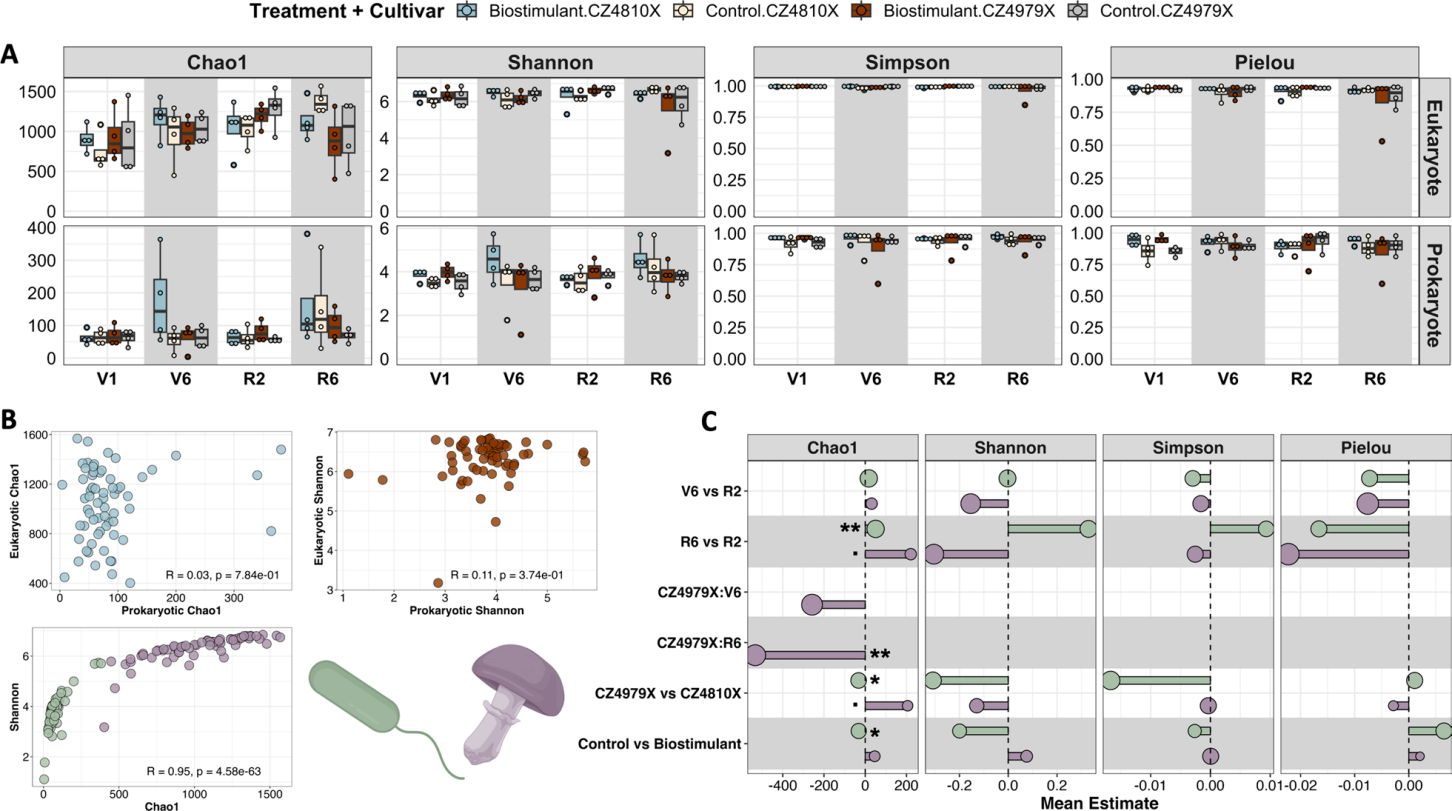
α diversity estimation. **A** Boxplots of the Chao1 index, Shannon diversity, Simpson diversity, and Pielou's evenness for eukaryotic and prokaryotic datasets. **B** Rank-based association between eukaryotic and prokaryotic Chao1 (top left), eukaryotic and prokaryotic Shannon diversity (top right), and Shannon diversity and Chao1 (bottom left). **C** Mean estimates (coefficients) for explanatory variables in α diversity GLMMs. Following removal of the baseline timepoint (V1), GLMMs were implemented to determine the effect of treatment, cultivar, and growth stage (fixed effects) on each response variable. Point size corresponds to hierarchically partitioned R^2^ values.^.^*p* ≤ 0.1, *p ≤ 0.05, **p ≤ 0.01, ***p ≤ 0.001

In assessing microbiome complexity and structure, the within-sample characteristics of ASV richness, diversity, and evenness were determined and compared across treatments, cultivars, and growth stages (Fig. 2A–C). ASV richness was measured with the non-parametric estimator Chao1 given its capacity to project undetected taxa based on the abundance of those rarely observed in a dataset. This extrapolated measure accounts for potential undersampling in high-diversity environments (e.g., soil), providing a more thorough assessment of community richness [49, 108]. Herein, the mean eukaryotic Chao1 index value was 1,033 ± 36, and was increased marginally in growth stage R6 vs R2 (*p*-value = 0.07, ME = 220.62) and in cultivar CZ4979X vs CZ4810X (*p*-value = 0.09, ME = 205.38) (Fig. 2C). A cultivar-growth stage interaction was also observed and explained the highest proportion of variance (marginalized R^2^ = 0.88), with CZ4979X:R6 being significantly less than the reference (*p*-value = 0.0023, ME = -533.75) (Fig. 2C). The prokaryotic Chao1 index was 85.44 ± 8.76 and demonstrated a significant increase in R6 vs R2 (*p*-value = 0.0099, ME = 50.28) and significant decreases in CZ4979X vs CZ4810X (*p*-value = 0.04, ME = -32.24) and in treatment control vs biostimulant (*p*-value = 0.04, ME = -31.72) (Fig. 2C). Moreover, growth stage explained the highest proportion of variance for prokaryotic Chao1 (marginalized R^2^ = 0.45). The baseline (V1) sampling showed no significant trends in the eukaryotic or prokaryotic Chao1 indices.

Shannon and Simpson indices were estimated to comprehensively assess α diversity. The Shannon index integrates ASV richness and evenness, with increasing values indicative of greater diversity and uniformity among ASV abundances [51]. Conversely, the Simpson index quantifies ASV dominance, where elevated values denote diminished dominance and heightened diversity [50]. The two indices displayed cooperative, statistically insignificant trends across fixed effect levels in the present study. Eukaryotic α diversity (6.3 ± 0.07 Shannon and 0.99 ± 0.002 Simpson) peaked at the R2 growth stage (evidenced by ME at V6 vs R2 and R2 vs R6), was reduced in CZ4979X vs CZ4810X, and was increased in control vs biostimulant, with growth stage explaining the highest proportion of variance (marginalized R^2^ = 0.73 and 0.33 for Shannon and Simpson indices, respectively) (Fig. 2C). The prokaryotic α diversity indices (3.82 ± 0.09 Shannon and 0.94 ± 0.007 Simpson) demonstrated an opposing trend, increasing with time and being reduced in control vs biostimulant (Fig. 2C). The direction of change between CZ4979X and CZ4810X was consistent with eukaryotic α diversity, and variance was most attributed to cultivar for both indices (marginalized R^2^ = 0.41 and 0.58 for Shannon and Simpson, respectively). Notably, statistically significant differences were detected in the baseline between control plots and those to which biostimulants would be applied, with the control plots displaying reduced eukaryotic and prokaryotic α diversity (eukaryotic Shannon *p*-value = 0.1, ME = -0.19; eukaryotic Simpson *p*-value = 0.05, ME = -0.001; prokaryotic Shannon *p*-value = 0.005, ME = -0.41; prokaryotic Simpson *p*-value = 0.002, ME = -0.04).

While α diversity incorporates both richness and evenness, relying solely on composite diversity indices might obscure their individual contributions to ecosystem function [109]. Pielou's evenness was therefore employed to quantify the count distribution across ASVs. The index was consistent between fixed effect levels and domains, with a mean of 0.91 ± 0.007 for eukaryotes, 0.90 ± 0.009 for prokaryotes, and no statistically significant trends observed (Fig. 2A, C). This observation was supported by a strong association between Chao1 richness and Shannon diversity (*ρ* = 0.95, *p*-value = 4.58e^−63^) (Fig. 2B). Eukaryotic evenness was influenced predominantly by growth stage (marginalized R^2^ = 0.98), while no patterns were present for the prokaryotic dataset. Consistent with Shannon and Simpson diversity, the baseline control plots showed reduced prokaryotic evenness in comparison to biostimulant plots (*p*-value = 0.0004, ME = -0.089).

**Fig. 3:**
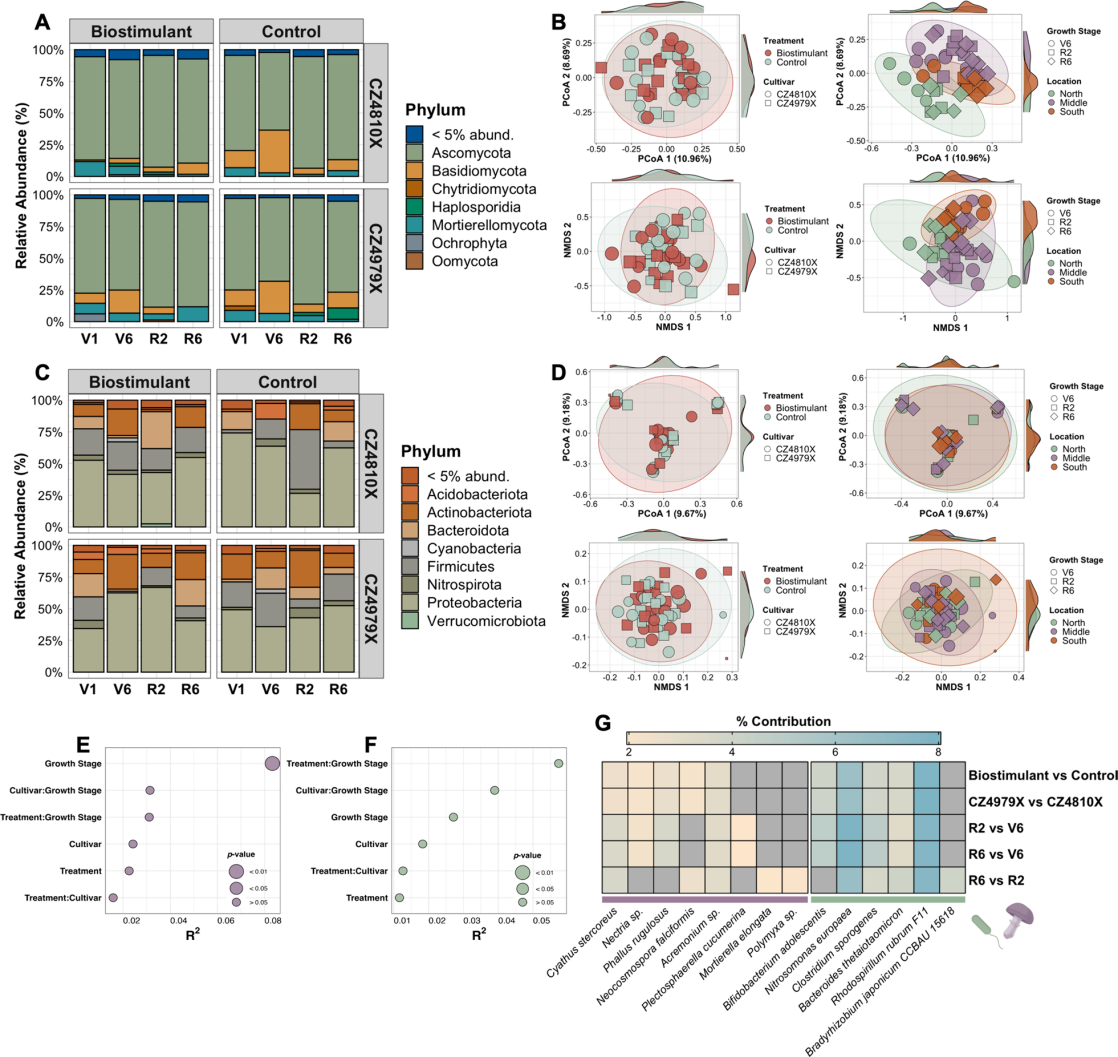
Microbiome composition and β diversity. **A** Relative abundance of eukaryotic phyla for each experimental condition. **B** PCoA (top row) and NMDS (bottom row) ordinations of eukaryotic Bray–Curtis dissimilarity. Compositional dissimilarity was calculated independently at ASV level using Bray–Curtis, Euclidean, and Jaccard distances, each of which yielded results consistent with those presented. Point size reflects Shannon diversity. **C** Relative abundance of prokaryotic phyla for each sample and experimental condition. **D** PCoA (top row) and NMDS (bottom row) ordinations of prokaryotic Bray–Curtis dissimilarity. Point size reflects Shannon diversity. **E** Variance explained by treatment, cultivar, growth stage, and interactions thereof on eukaryotic community composition as determined by PERMANOVA with Bray–Curtis dissimilarity. **F** Variance explained for prokaryotic community composition. **G** Heatmap of ASVs most influential for pairwise dissimilarity between fixed effect levels. The lowest taxonomic classification for each ASV is displayed below the corresponding column

Compositional dissimilarity was first assessed using CSS-normalized counts from full-length, mapped ASVs. The phylum-level composition was first visualized across experimental conditions, revealing that the eukaryotic rhizosphere microbiome was largely composed of Ascomycota and the prokaryotic microbiome dominated by Proteobacteria (Fig. 3A, C). Dissimilarity matrices were constructed subsequently leveraging Bray–Curtis, Euclidean, and Jaccard distances, and compositional trends were inferred between fixed effect levels with PERMANOVA and β dispersion estimation. Findings were consistent across the matrices (Supplementary Table 1, 2, Additional file 2); therefore, representative ordinations were generated with Bray–Curtis dissimilarity (Fig. 3B, D). Notably, growth stage had the most significant impact on eukaryotic microbiome composition (Bray–Curtis PERMANOVA R^2^ = 0.08, *F*-value = 2.0, *p*-value = 0.0001) (Fig. 3E; Supplementary Table 1, Additional file 2), yet ANOVA suggested heterogeneous β dispersion across growth stage levels (*F*-value = 6.32, *p*-value = 0.003) (Supplementary Table 1, Additional file 2). Both PCoA and NMDS ordinations implicated a strong spatial effect on eukaryotic microbiome composition as well (Fig. 3B). Prokaryotic microbiome composition showed neither strong statistical nor qualitative trends, albeit a treatment-growth stage interaction explained the highest proportion of variance (Bray–Curtis PERMANOVA R^2^ = 0.06, *F*-value = 1.31, *p*-value = 0.08) (Fig. 3D, F; Supplementary Table 2, Additional file 2). Lastly, while no compositional trends emerged from the baseline measurements, β dispersion did vary between treatment levels for Euclidean dissimilarity (ANOVA *F*-value = 4.48, *p*-value = 0.05) (Supplementary Fig. 2; Supplementary Table 3, Additional file 2).

In like manner, Bray–Curtis dissimilarity matrices were reconstructed with CSS-normalized counts from complete datasets (both mapped and unmapped ASVs), and compositional trends were assessed. Consistent with the prior analysis, growth stage explained the most variance in eukaryotic compositional dissimilarity (PERMANOVA R^2^ = 0.05, *F*-value = 1.10,* p*-value = 0.003), and β dispersion was not significantly heterogeneous among effect levels (Supplementary Table 4, Additional file 2). In addition, treatment had a statistically significant effect on composition (PERMANOVA *F*-value = 1.10, *p*-value = 0.02), while cultivar demonstrated a marginally significant effect (PERMANOVA *F*-value = 1.03, *p*-value = 0.07) (Supplementary Table 4, Additional file 2). Regarding prokaryotic compositional dissimilarity, growth stage explained the most variance (PERMANOVA R^2^ = 0.05, *F*-value = 1.19, *p*-value = 0.02) rather than a treatment-growth stage interaction as observed with the reduced dataset, yet demonstrated heterogeneous β dispersion (*F*-value = 3.25, *p*-value = 0.05) (Supplementary Table 4, Additional file 2). The treatment-growth stage interaction significantly influenced prokaryotic compositional dissimilarity (PERMANOVA *F*-value = 1.11, *p*-value = 0.08) (Supplementary Table 4, Additional file 2). No statistical significance was observed in baseline measurements of eukaryotic or prokaryotic β diversity (Supplementary Table 5, Additional file 2).

Complementary to PERMANOVA, the Bray–Curtis indices derived from mapped ASVs were decomposed with the Similarity Percentage method [71] to discern ASVs most influential for pairwise similarity/dissimilarity between fixed effect levels. The five ASVs to which dissimilarity was most attributed were identified for each comparison, and their lowest taxonomic classification retrieved. Interestingly, *Cyathus stercoreus* and an *Acremonium* species were identified for all five fixed effect comparisons with the eukaryotic Bray–Curtis matrix, followed by a *Nectria* species and *Phallus rugulosus* in four comparisons, and *Neocosmospora falciformis* in three. *Plectosphaerella cucumerina* was exclusive to comparisons between vegetative and reproductive growth stages, while *Mortierella elongata* and a *Polymyxa* species were exclusive to R6 vs R2 (Fig. 3G). Likewise, ASVs corresponding to *Nitrosomonas europaea*, *Clostridium sporogenes*, *Bacteroides thetaiotaomicron*, and *Rhodospirillum rubrum* F11 were most influential for all fixed effect comparisons of prokaryotic Bray–Curtis dissimilarity, with *Bifidobacterium adolescentis* identified in four comparisons and *Bradyrhizobium japonicum* CCBAU 15618 exclusive to R6 vs R2 (Fig. 3G). Given the vast overlap of these ASVs between pairwise comparisons, and that all demonstrated statistical insignificance (*p*-value > 0.05 based on 9,999 permutations), ASV identification more likely reflected high abundance/variation across the amplicon datasets than contribution to dissimilarity, which is a common (yet often misinterpreted) element of Similarity Percentage analysis [71].

### Microbiota with agriculturally-relevant life strategies exhibited distinct membership trends across fixed effect levels

**Fig. 4:**
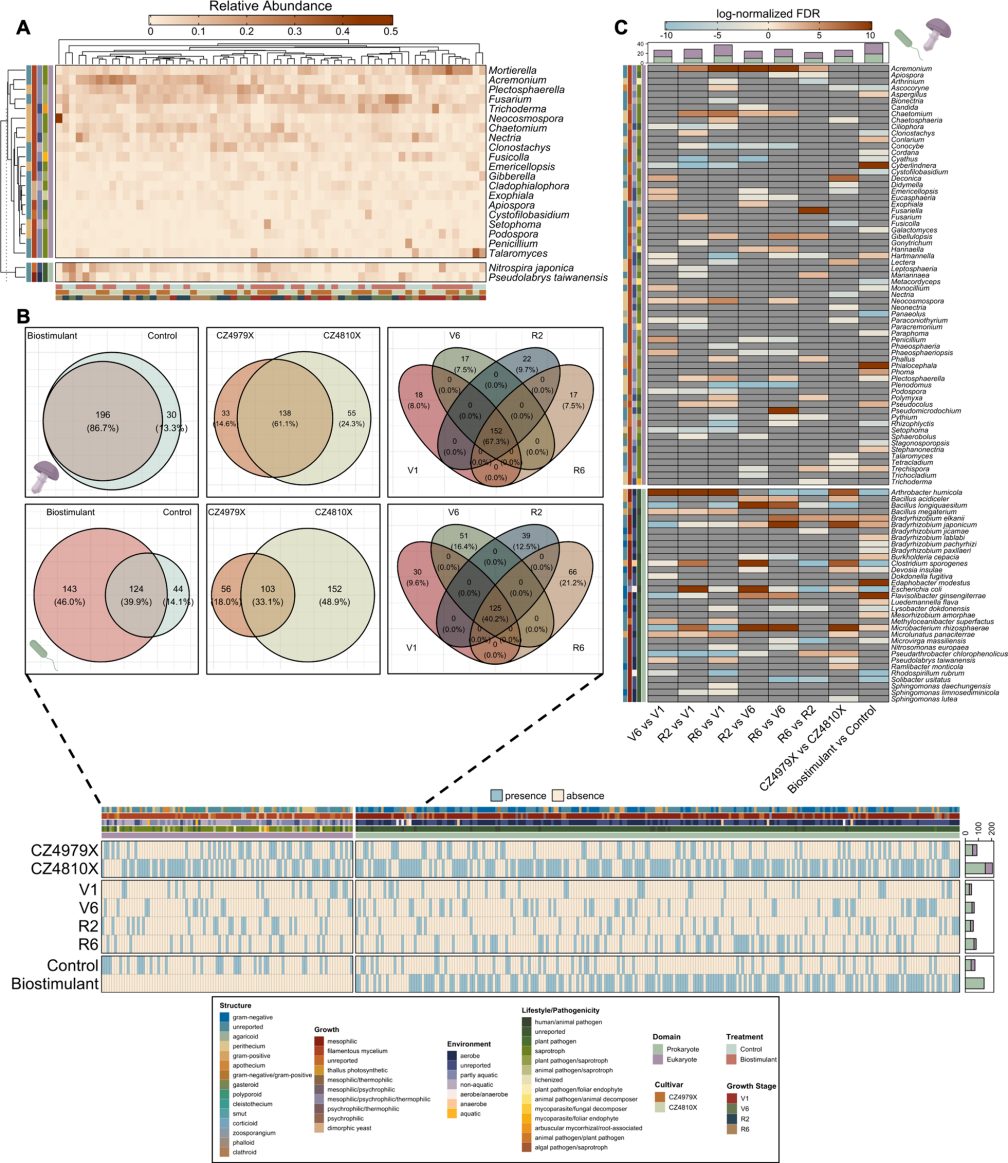
Community membership analyses. Community membership was determined at genus and species levels for eukaryotic and prokaryotic communities, respectively, which were the lowest taxonomic classifications to which 100% ASVs mapped. **A** Core taxa demonstrating a prevalence ≥ 0.5 across all samples. Left heatmap annotations are taxon metadata, and bottom annotations are sample metadata. **B** The number of shared and unique taxa by treatment (left), cultivar (middle), and growth stage (right) for eukaryotic (top) and prokaryotic (bottom) communities. The corresponding heatmap displays presence/absence of unique taxa across fixed effect levels (summarized collectively and by domain in left bar plots) in addition to taxon metadata (top annotation). **C** Differentially abundant taxa between experimental conditions. Left heatmap annotations are taxon metadata, and top annotation is the number of differentially abundant taxa (summarized collectively and by domain) between fixed effect levels (bottom). Eukaryotes are purple and prokaryotes are green for all bar plot annotations

Core, unique, and differentially abundant taxa were identified at genus and species levels for eukaryotes and prokaryotes, respectively, across all growth stages (baseline and those succeeding biostimulant application). Unsurprisingly, 20/22 core taxa (defined as those with a ≥ 0.5 prevalence across all samples) were eukaryotic genera that mostly exhibit a partly aquatic, saprotrophic lifestyle with filamentous mycelial growth and perithecial fruiting bodies (Fig. 4A). Furthermore, 7/20 (35%) were annotated as plant pathogens (Fig. 4A). The prokaryotic core members included the nitrite-oxidizing bacterium *Nitrospira japonica* [110] and the scarcely reported bacterium *Pseudolabrys taiwanensis* [111] (Fig. 4A).

Eukaryotic genera/prokaryotic species unique to a fixed effect level encompassed a collective 217 taxa between treatments (30 eukaryotes, 187 prokaryotes), 296 between cultivars (88 eukaryotes, 208 prokaryotes), and 260 between growth stages (74 prokaryotes, 186 eukaryotes) (Fig. 4B). Those unique to biostimulant-treated samples were all prokaryotes and included four species in the symbiotic genus *Bradyrhizobium* [112], the additional rhizobia *Mesorhizobium ciceri*, *Mesorhizobium plurifarium*, *Rhizobium grahamii*, and *Rhizobium massiliae*, *Pseudomonas fluorescens* [113], three species of *Bacillus* [114], and 10 *Streptomyces* species [115] (Fig. 4B). Notable taxa exclusive to the control treatment were the arbuscular mycorrhizal/root-associated genus *Paraglomus* [116], six plant pathogens (including the soybean disease-causing oomycete genus *Phytophthora* [117]), and *Bradyrhizobium stylosanthis* (Fig. 4B).

Perhaps the most distinct trend was the exclusivity of plant pathogens to a particular cultivar. Eight pathogens were unique to CZ4979X (SDS-tolerant cultivar), including *Phytophthora* and the soybean-parasitizing fungal genus *Septoria* [118] (Fig. 4B). Other CZ4979X-specific taxa were *Bradyrhizobium algeriense*, *Bradyrhizobium betae*, *Mesorhizobium plurifarium*, *Nitrospira multiformis* [119], and *Pseudomonas fluorescens* (Fig. 4B). Samples from CZ4810X, which presumably have heightened susceptibility to the soybean disease SDS in comparison to CZ4979X, had 15 plant pathogens not present in CZ4979X samples, including the soybean disease-causing genera *Diaporthe* [120], *Macrophomina* [121], and *Rhizoctonia* [122] (Fig. 4B). Additional taxa unique to CZ4810X included the genus *Paraglomus*, *Bradyrhizobium lupini*, *Bradyrhizobium stylosanthis*, *Mesorhizobium ciceri*, *Rhizobium cellulosilyticum*, and nine species of *Streptomyces*.

Of the 260 taxa exclusive to a single growth stage, 48 were unique to V1 (18 eukaryotes, 30 prokaryotes), 68 to V6 (17 eukaryotes, 51 prokaryotes), 61 to R2 (22 eukaryotes, 39 prokaryotes), and 83 to R6 (17 eukaryotes, 66 prokaryotes) (Fig. 4B). Those corresponding to V1 included six plant pathogens (including *Septoria*), *Nitrospira multiformis*, and *Bradyrhizobium algeriense* (Fig. 4B). The V6 growth stage was characterized by three distinct plant pathogens, *Paraglomus*, *Bradyrhizobium lupini*, two *Bacillus* species, and nine *Streptomyces* species (Fig. 4B). Similarly, R2 possessed five unique plant pathogens (including *Rhizoctonia*), *Bradyrhizobium betae*, *Mesorhizobium plurifarium*, and *Rhizobium cellulosilyticum* (Fig. 4B). The final sampled growth stage contained 5 unique plant pathogens (including *Macrophomina*, *Phytophthora*, and *Cercospora* [123]), two *Bacillus* species, *Bradyrhizobium stylosanthis*, and *Pseudomonas fluorescens* (Fig. 4B). A comprehensive list of condition-specific taxa can be found in Additional file 5.

Differentially abundant taxa between fixed effect levels were determined by fitting CSS-normalized counts with a ZINB regression model. The greatest number of those statistically enriched/depleted (*q*-value < 0.25) was observed between levels of treatment (22 eukaryotes, 19 prokaryotes, total *n* = 41), with 18 taxa being enriched and 23 depleted in biostimulant vs control (Fig. 4C). Notably, this included the differential abundance of saprotrophic fungi, the depletion of five fungal pathogens, and the enrichment of *Bradyrhizobium elkanii*, *Bradyrhizobium japonicum*, *Bradyrhizobium lablabi*, and *Mesorhizobium amorphae* (Fig. 4C). The most depleted taxa in biostimulant-treated samples were the potential human/foodborne pathogenic bacteria *Clostridium sporogenes* and *Escherichia coli* [124, 125] (Fig. 4C). Twenty-seven taxa (13 eukaryotes, 14 prokaryotes) were differentially abundant between cultivars, 14 of which were enriched and 13 depleted in CZ4979X vs CZ4810X (Fig. 4C). Most of the identified eukaryotes displayed marginal depletion in CZ4979X (including 3/4 differentially abundant plant pathogens) (Fig. 4C). In contrast, the majority of prokaryotes were enriched, including the inorganic phosphate-solubilizing bacterium *Bacillus acidiceler* [126], *Bradyrhizobium elkanii*, *Bradyrhizobium japonicum*, and *Escherichia coli* (Fig. 4C).

As expected, an increase in differentially abundant taxa was associated with the temporal distinctiveness of compared growth stages. The greatest number was observed in the R6 vs V1 comparison (distance = 3 growth stages) (23 eukaryotes, 15 prokaryotes, total *n* = 38), followed by R6 vs V6 (distance = 2 growth stages) (16 eukaryotes, 13 prokaryotes, total *n* = 29), R2 vs V1 (distance = 2 growth stages) (19 eukaryotes, 10 prokaryotes, total *n* = 29), V6 vs V1 (distance = 1 growth stage) (14 eukaryotes, 13 prokaryotes, total *n* = 27), R2 vs V6 (distance = 1 growth stage) (15 eukaryotes, 10 prokaryotes, total *n* = 25), and R6 vs R2 (distance = 1 growth stage) (12 eukaryotes, 10 prokaryotes, total *n* = 22) (Fig. 4C). The eukaryotic dataset was defined by the enrichment of saprotrophic genera *Acremonium* and *Chaetomium* with the progression of time, which was particularly distinct between vegetative and reproductive growth, and a depletion in the saprotrophic genus *Conocybe* (Fig. 4C). The reproductive growth stages also displayed an overall enrichment in *Microbacterium rhizosphaerae*, *Bradyrhizobium elkanii,* and *Bradyrhizobium japonicum*, although the latter was depleted at all stages compared to V1 (Fig. 4C). To this end, the V1 growth stage showed unique enrichment of eukaryotic genera *Ciliophora*, *Cyberlindnera*, *Podospora*, and *Setophoma*, of prokaryotic species *Bradyrhizobium japonicum* and *Pseudarthrobacter chlorophenolicus*, and a depletion in the genus *Neocosmospora* and species *Arthrobacter humicola*, *Bacillus megaterium*, and *Microlunatus panaciterrae* (Fig. 4C). Differentially abundant taxa and associated metadata are provided in Additional file 6.

### A treatment-cultivar interaction defined genus-level microbial co-occurrence network structure

**Fig. 5:**
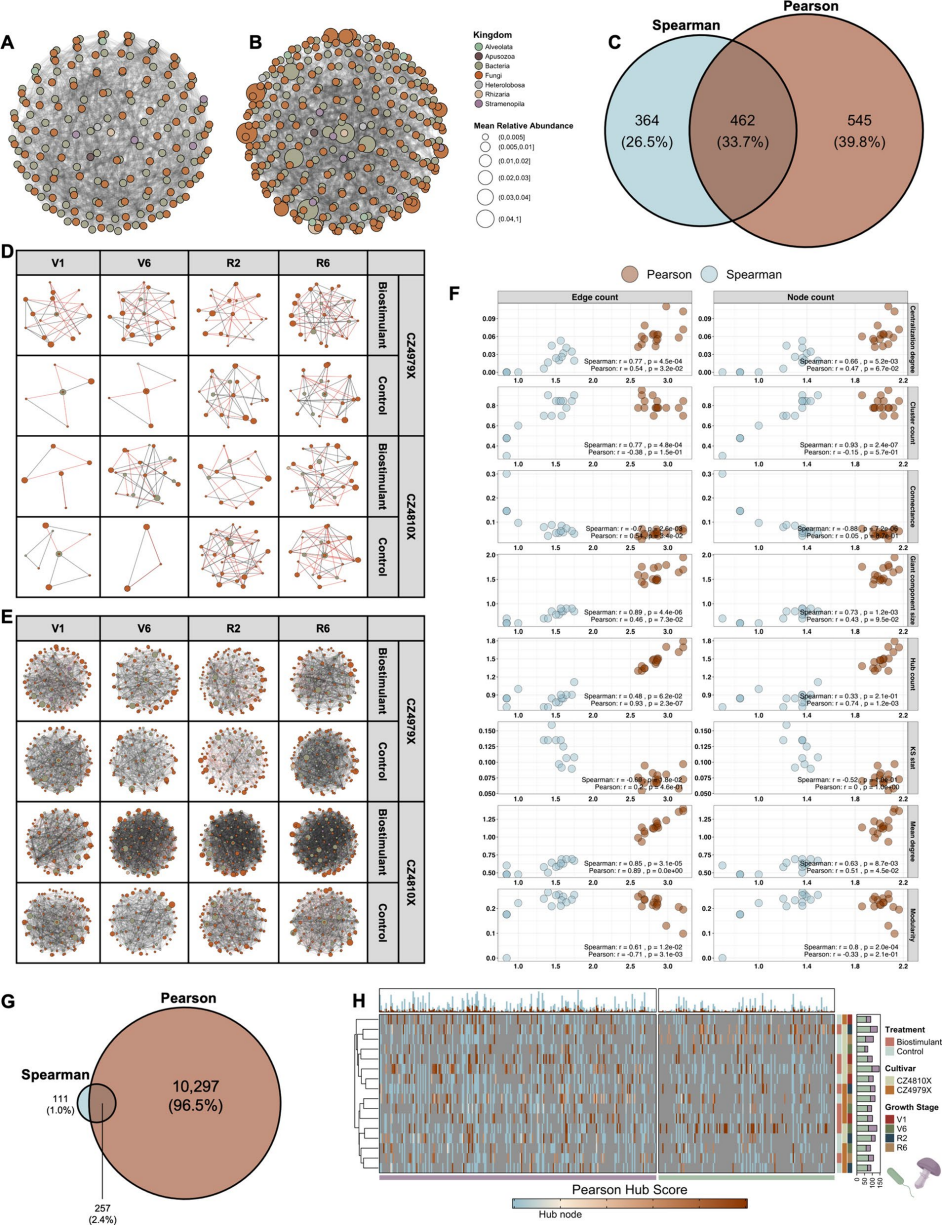
Global and condition-specific co-occurrence network analysis. **A** Global genus-level co-occurrence network constructed by obtaining significant positive and negative pairwise Spearman associations (Rho >  ± 0.6, *q*-value ≤ 0.05). **B** Global genus-level co-occurrence network constructed by obtaining significant positive and negative Pearson associations. **C** Venn diagram of unique and overlapping co-occurrences between Spearman and Pearson global networks. **D** Condition-specific networks constructed with significant Spearman associations. **E** Condition-specific networks constructed with significant Pearson associations. **F** Spearman associations between network density (edge count and node count) and topological features for each set of condition-specific networks. Both x and y axes represent log_10_ values. **G** Venn diagram of unique and overlapping co-occurrences between Spearman and Pearson condition-specific networks. (**H**) Heatmap of Pearson/Spearman condition-specific network nodes. Node color represents Kleinberg hub centrality, with blue reflecting a network member (hub score < 0.2) and tan/red reflecting a network hub (hub score ≥ 0.2). The top annotation represents the number of networks in which a node is a network member (blue) or hub (red). The right annotation shows the number of genera in each condition-specific network and is partitioned by domain (eukaryotes are purple and prokaryotes are green)

Putative genus-genus associations were inferred with Spearman and Pearson correlation methods. The associations were determined from CSS-normalized absolute abundances to mitigate the concomitant limitations of compositionality bias and biases stemming from differential sampling efficiency of taxa [127, 128]. Moreover, the *p*-values of pairwise associations were corrected for multiple testing given the prevalence of Type I errors during microbial co-occurrence network construction [91]. Herein, the global Spearman co-occurrence network comprised 826 edges (associations) and 188 nodes (genera), all of which presented a low mean relative abundance (< 0.5%) across the dataset (Fig. 5A, C). The global Pearson network possessed 1007 edges and 294 nodes, with evident variability in mean relative abundance observed (Fig. 5B, C). In addition, 462/1,371 (33.7%) of the edges were shared between the networks (Fig. 5C).

Condition-specific co-occurrence networks, defined by treatment-cultivar-growth stage combinations, were constructed with Spearman and Pearson associations as outlined for global networks. The Spearman networks presented an increase in network density with time (vegetative node *n* = 11.13 ± 2.54, edge *n* = 16.38 ± 4.88; reproductive node *n* = 22.88 ± 1.51, edge *n* = 37.50 ± 4.27), and a potential treatment effect at the V6 growth stage (control node *n* = 5.0 ± 1.0, edge *n* = 6; biostimulant node *n* = 21.0 ± 2.0, edge *n* = 35.50 ± 1.50) (Fig. 5D; Table 1). Given the density of the biostimulant-CZ4979X-V1 network (node *n* = 16, edge *n* = 27), which is the baseline sampling preceding biostimulant application, the variation observed at V6 may better reflect a treatment-cultivar interaction between biostimulant and CZ4810X (Fig. 5D; Table 1). In support of this notion, the greatest trend in Pearson network density was observed in biostimulant-CZ4810X networks at growth stages succeeding biostimulant application (control node *n* = 99.0 ± 14.42, edge *n* = 612.33 ± 86.81; biostimulant node *n* = 136.0 ± 4.58, edge *n* = 1,533.67 ± 67.85) (Fig. 5E; Table 2). Pearson networks were overall denser than Spearman networks (Spearman node *n* = 17.0 ± 2.08, edge *n* = 26.94 ± 4.15; Pearson node *n* = 107.63 ± 4.78, edge *n* = 791.50 ± 99.20), which was consistent with the global co-occurrence networks, and 257/10,665 (2.41%) of edges were shared between the association methods (Fig. 5E, G).

**Table 1:** Topological properties for condition-specific Spearman networks

Network	Centralization degree	Cluster count	Connectance	Edge count	Giant component size	Hub count	KS stat	Mean degree	Modularity	Node count
V1-Bst-79X	0.11	4.00	0.23	27.00	6.00	6.00	0.44	3.38	0.62	16.00
V6-Bst-79X	0.06	4.00	0.22	37.00	6.00	6.00	0.23	3.89	0.71	19.00
R2-Bst-79X	0.05	7.00	0.13	55.00	6.00	12.00	0.28	3.67	0.80	30.00
R6-Bst-79X	0.04	7.00	0.10	24.00	4.00	4.00	0.36	2.18	0.84	22.00
V1-Ctrl-79X	0.00	2.00	0.40	6.00	3.00	6.00	*NA*	2.00	0.50	6.00
V6-Ctrl-79X	0.00	2.00	0.40	6.00	3.00	6.00	*NA*	2.00	0.50	6.00
R2-Ctrl-79X	0.06	6.00	0.13	31.00	5.00	5.00	0.36	2.82	0.79	22.00
R6-Ctrl-79X	0.13	6.00	0.16	36.00	7.00	7.00	0.36	3.27	0.63	22.00
V1-Bst-10X	0.00	2.00	0.40	6.00	3.00	6.00	*NA*	2.00	0.50	6.00
V6-Bst-10X	0.05	6.00	0.13	34.00	5.00	5.00	0.25	2.96	0.80	23.00
R2-Bst-10X	0.01	4.00	0.20	21.00	4.00	4.00	0.36	2.80	0.73	15.00
R6-Bst-10X	0.08	6.00	0.14	39.00	6.00	6.00	0.33	3.25	0.77	24.00
V1-Ctrl-10X	0.00	3.00	0.25	9.00	3.00	9.00	*NA*	2.00	0.67	9.00
V6-Ctrl-10X	0.00	1.00	1.00	6.00	4.00	4.00	*NA*	3.00	0.00	4.00
R2-Ctrl-10X	0.08	6.00	0.16	51.00	7.00	7.00	0.23	3.92	0.72	26.00
R6-Ctrl-10X	0.10	5.00	0.19	43.00	7.00	7.00	0.28	3.91	0.68	22.00

Column 1: Growth Stage-Treatment-Cultivar; Ctrl = Control; Bst = Biostimulant; 79X = CZ4979X; 10X = CZ4810X

**Table 2:** Topological properties for condition-specific Pearson networks

Network	Centralization degree	Cluster count	Connectance	Edge count	Giant component size	Hub count	KS stat	Mean degree	Modularity	Node count
V1-Bst-79X	0.16	7.00	0.12	627.00	31.00	29.00	0.21	12.29	0.64	102.00
V6-Bst-79X	0.20	6.00	0.11	489.00	36.00	21.00	0.24	10.19	0.72	96.00
R2-Bst-79X	0.11	8.00	0.06	447.00	27.00	19.00	0.13	7.58	0.81	118.00
R6-Bst-79X	0.16	6.00	0.12	719.00	32.00	31.00	0.17	13.31	0.67	108.00
V1-Ctrl-79X	0.13	5.00	0.17	700.00	30.00	27.00	0.17	15.56	0.61	90.00
V6-Ctrl-79X	0.14	5.00	0.13	485.00	24.00	21.00	0.18	11.02	0.73	88.00
R2-Ctrl-79X	0.10	5.00	0.10	399.00	38.00	19.00	0.19	8.77	0.77	91.00
R6-Ctrl-79X	0.29	5.00	0.14	951.00	61.00	49.00	0.20	16.12	0.35	118.00
V1-Bst-10X	0.14	5.00	0.15	725.00	29.00	29.00	0.20	14.50	0.64	100.00
V6-Bst-10X	0.14	5.00	0.17	1,398.00	43.00	40.00	0.14	21.51	0.61	130.00
R2-Bst-10X	0.26	4.00	0.18	1,599.00	88.00	61.00	0.25	24.05	0.25	133.00
R6-Bst-10X	0.18	6.00	0.15	1,604.00	49.00	48.00	0.18	22.12	0.57	145.00
V1-Ctrl-10X	0.11	5.00	0.12	684.00	29.00	25.00	0.18	12.91	0.74	106.00
V6-Ctrl-10X	0.15	7.00	0.18	448.00	56.00	23.00	0.18	12.62	0.62	71.00
R2-Ctrl-10X	0.16	5.00	0.11	743.00	56.00	31.00	0.18	12.49	0.73	119.00
R6-Ctrl-10X	0.16	4.00	0.11	646.00	53.00	30.00	0.17	12.07	0.68	107.00

Column 1: Growth Stage-Treatment-Cultivar; Ctrl = Control; Bst = Biostimulant; 79X = CZ4979X; 10X = CZ4810X

Condition-specific network topology was further defined by centralization degree (the extent to which a single node “controls” a network), cluster count (the number of separate, interconnected groups), connectance (the proportion of all possible links that are actual connections), giant component size (the size of the largest connected subgraph), hub count (the number of nodes with a Kleinberg hub centrality score > 0.2), KS test statistic (how closely degree distribution adheres to a scale-free topology), mean node degree (the average number of connections per node), and modularity (the strength of division into distinct modules/communities) (Tables 1, 2). Of these, centralization degree, cluster count, giant component size, mean degree, and modularity demonstrated a statistically significant, positive association (Spearman’s ρ statistic > 0, *p*-value < 0.05) with both edge count and node count across Spearman networks, while connectance presented a negative association (Fig. 5F; Table 1). The KS test statistic was negatively associated with edge count (Fig. 5F; Table 1). Conversely, only hub count and mean degree presented significant associations with Pearson network node count (both of which were positive), and centralization degree, connectance, hub count, mean degree, and modularity were associated with edge count (all positive associations except modularity) (Fig. 5F; Table 2). The discrepancy in network topology was reflected in the ranking of co-occurrence networks, as eight of the 10 metrics showed statistically significant differences in conditional ranking between the association methods (Friedman rank sum test *p* < 0.05) (Supplementary Fig. 3B, Additional file 2). Despite this, all nodes represented in Spearman networks were present in Pearson networks (Supplementary Fig. 3A, Additional file 2), and the hierarchical clustering of Pearson networks by node centrality score supported the overarching trend of treatment-cultivar interaction defining co-occurrence network structure (Fig. 5H).

### Edaphic property dynamism and integration within phenotype-taxon networks

**Fig. 6:**
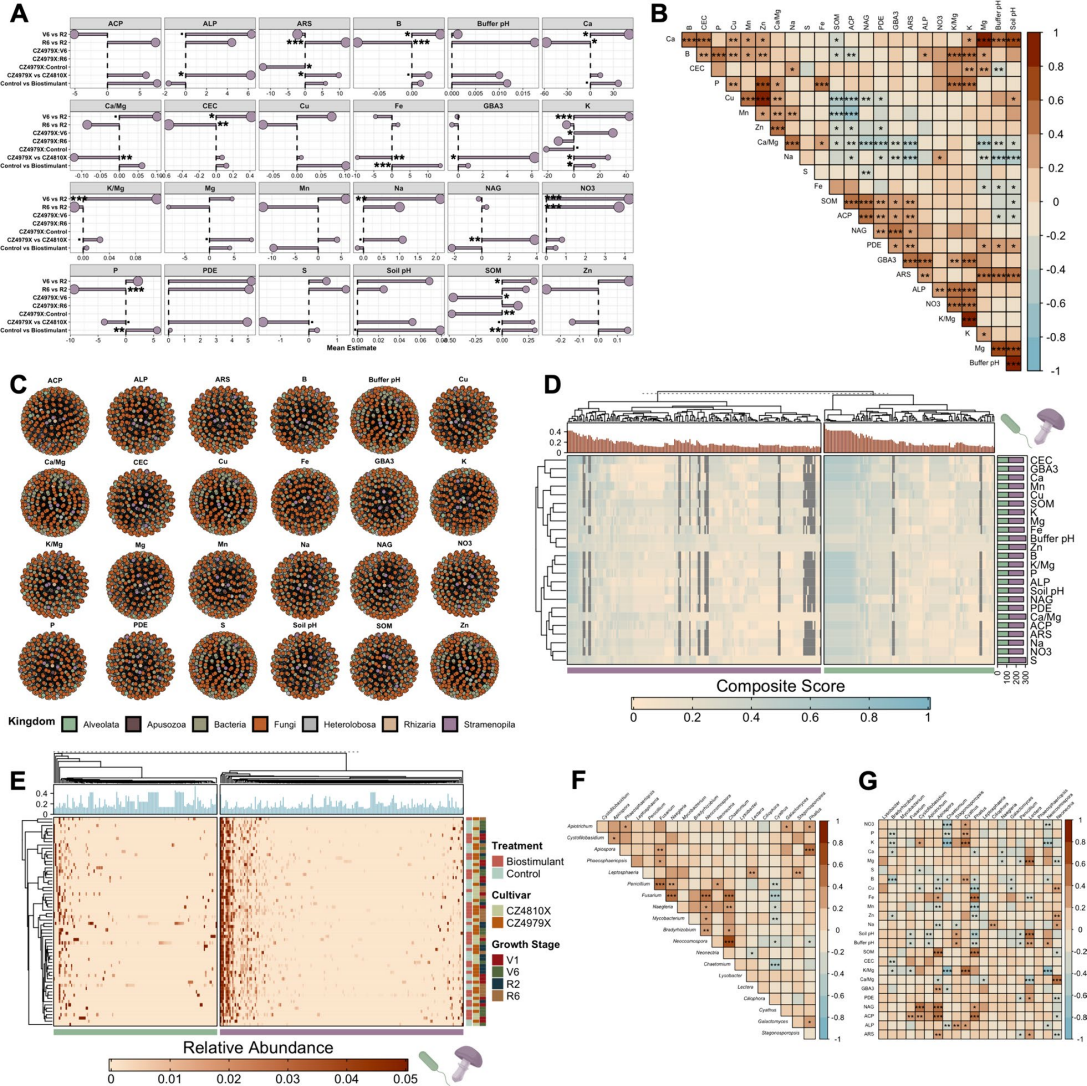
Phenotype-taxon networks for edaphic parameters. **A** Mean estimates for edaphic measure GLMMs. Point size corresponds to hierarchically partitioned R^2^ values. **B** Pairwise Spearman associations for edaphic measures. **C** Genus-level phenotype-taxon networks constructed by coupling lasso regression, reduced GLMs, and co-occurrences (all significant Spearman and Pearson associations). **D** Heatmap of node composite score (calculated with normalized modularity measures and Kleinberg's hub centrality) for each phenotype-taxon network. The top annotation represents mean composite score across all networks. The right annotation shows the number of nodes in each phenotype-taxon network and is partitioned by domain (eukaryotes are purple and prokaryotes are green). **E** Relative abundance of nodes in phenotype-taxon networks. The top annotation represents the mean composite score. **F** Pairwise Spearman associations for the top 20 nodes with respect to mean composite score. **G** Pairwise Spearman associations for the top 20 nodes and edaphic measures.^.^*p* ≤ 0.1, *p ≤ 0.05, **p ≤ 0.01, ***p ≤ 0.001

To contextualize microbiome dynamics, 24 edaphic parameters were measured for each soil sample, and differences between fixed effect levels and their interactions were determined using GLMMs. Of these, 18 parameters displayed significant variation (*p* < 0.05) between levels of one or more explanatory variables, with soil K and SOM affected most (*n* = 5). These were followed by ARS, B, Ca, and P (*n* = 3), and ALP, Ca/Mg, CEC, Fe, K/Mg, Na, and NO_3_- (*n* = 2). The parameters GBA3, Mg, NAG, S, and soil pH each displayed variation between levels of a single explanatory variable (Fig. 6A). Inversely, cultivar explained observed variation for 14 edaphic parameters, 12 of which were increased in CZ4979X vs CZ4810X (Fig. 6A). Ten parameters were increased in V6 vs R2, and six differed significantly in the R6 vs R2 comparison (two increased and four decreased) (Fig. 6A). The explanatory variables treatment, treatment-cultivar interaction, and cultivar-growth stage interaction each explained observed variation in a lesser number of edaphic parameters (Fig. 6A). Furthermore, rank-based measures of association were inferred between edaphic parameters with Spearman’s ρ statistic, rendering 109 significant associations (positive *n* = 73, negative *n* = 36) (Fig. 6B). Of these, the Ca/Mg ratio presented the most significant associations of the parameters (positive *n* = 6, negative *n* = 9; total *n* = 15) (Fig. 6B).

Significant microbial associations spanning one or both global networks were coupled with edaphic data to construct phenotype-taxon networks. Briefly, lasso regression was used to identify taxa putatively associated with each edaphic parameter given its propensity to assign coefficient penalties in instances when sample size is small relative to feature (node) count [89, 129]. A reduced GLM was deployed thereafter to provide directionality to phenotype-node associations and was overlaid with the global microbial association dataset to derive final networks. The phenotype-taxon networks comprised 285.63 ± 1.98 nodes (eukaryotic *n* = 165.71 ± 1.70, prokaryotic *n* = 119.92 ± 0.31) and 1,417.46 ± 24.33 edges, and a collective 306 nodes (183 eukaryotes, 123 prokaryotes) were represented in at least one network (Fig. 6C, D). Given the inherent zero inflation in taxon abundance (Fig. 6E), node prioritization was preceded by filtering to those with a ≥ 0.2 prevalence. A composite score was then computed with modularity measures and Kleinberg's hub centrality (see “Materials and Methods”), and the top 20 nodes by mean composite score were extracted. These nodes included 17 eukaryotic genera that are predominantly aquatic/partly aquatic with mycelial growth and a saprotrophic lifestyle, as well as the prokaryotic genera *Bradyrhizobium*, *Lysobacter*, and *Mycobacterium* (Fig. 6F, G). Six of the 20 (30%) were represented in the core microbiome. In addition, seven (35%) of the selected eukaryotic genera were annotated as plant pathogens.

Rank-based measures of association were determined thereafter between prioritized nodes, as well as between nodes and edaphic phenotypes. Thirty node–node associations were significant (positive *n* = 22, negative *n* = 8), with the greatest number of those encompassing the pathogenic/saprotrophic genus *Fusarium* (positive *n* = 6, negative *n* = 1, total *n* = 7) and the saprotrophic genus *Neocosmospora* (positive *n* = 5, negative *n* = 2, total *n* = 7) (Fig. 6F). The node-phenotype analysis rendered 90 significant associations (positive *n* = 34, negative *n* = 56), with the most represented nodes being the pathogenic *Apiospora* (positive *n* = 6, negative *n* = 5, total *n* = 11) and the saprotrophic *Phallus* (positive *n* = 4, negative *n* = 7, total *n* = 11), and the most represented phenotypes being B (positive *n* = 1, negative *n* = 6, total *n* = 7) and Buffer pH (positive *n* = 3, negative *n* = 4, total *n* = 7) (Fig. 6G).

### Agronomic property dynamism and integration within phenotype-taxon networks

**Fig. 7:**
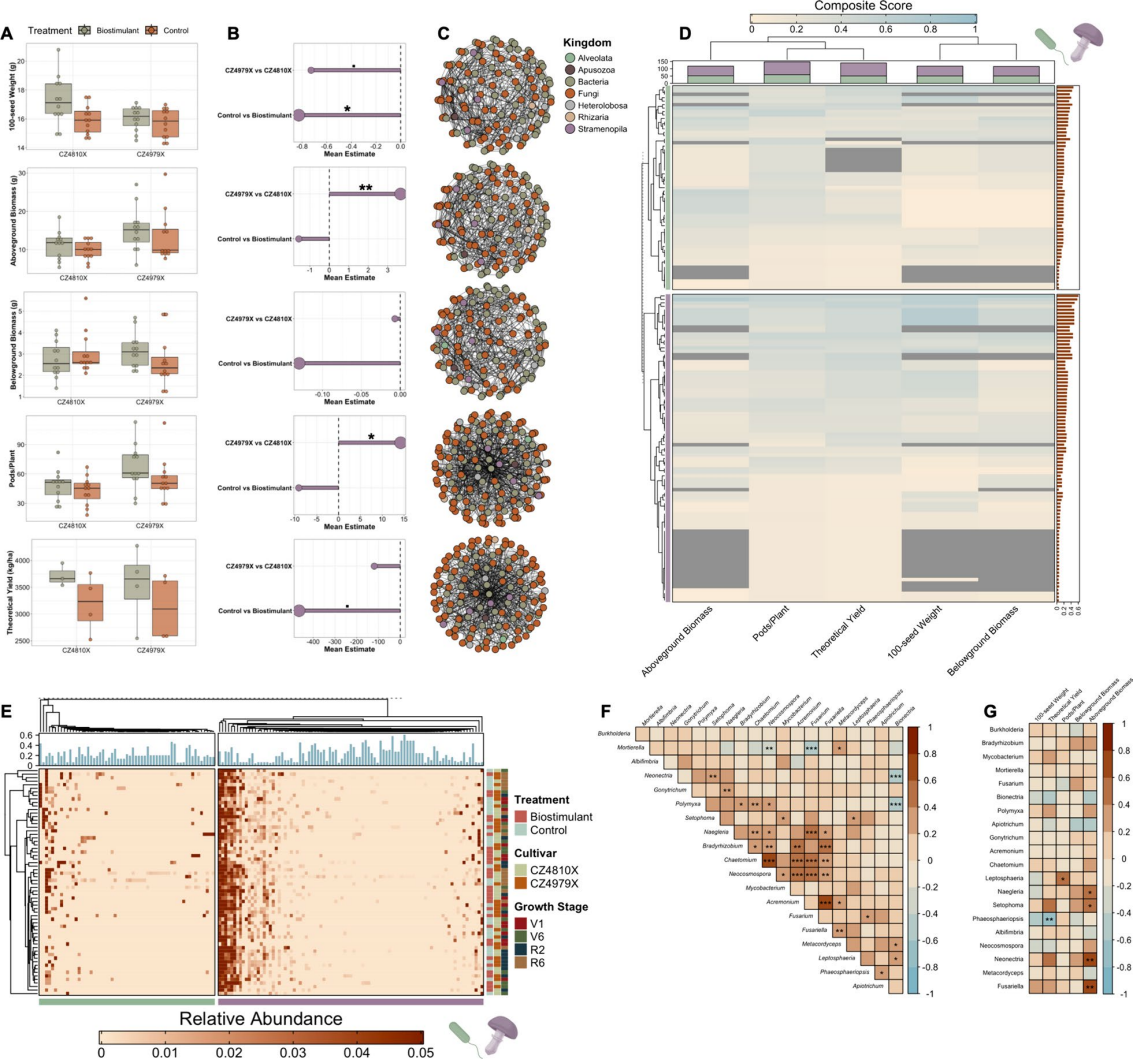
Phenotype-taxon networks for agronomic parameters. **A** Agronomic measures across treatments and cultivars. Note that biomass measurements reflect dry weight, and 100-seed weight and theoretical yield were determined at 13% moisture. **B** Mean estimates for agronomic measure GLMMs. (**C**) Genus-level phenotype-taxon networks. **D** Heatmap of node composite score for each phenotype-taxon network. The right annotation shows the mean composite score across all networks for each node. The top annotation shows the number of nodes in each phenotype-taxon network and is partitioned by domain (eukaryotes are purple and prokaryotes are green). **E** Relative abundance of nodes in phenotype-taxon networks. The top annotation shows the mean composite score. **F** Pairwise Spearman associations for the top 20 nodes with respect to composite score. **G** Pairwise Spearman associations for the top 20 nodes and agronomic measures.^.^*p* ≤ 0.1, *p ≤ 0.05, **p ≤ 0.01, ***p ≤ 0.001

At the R8 growth stage, the agronomic parameters 100-seed weight, aboveground biomass, belowground biomass, pods/plant, and theoretical yield were determined for each of the 16 plots. Variations between levels of treatment, cultivar, and their interaction were then determined using GLMMs. Biostimulant-treated plots had significantly increased 100-seed weight (*p*-value = 0.05, ME = 0.83), and a marginal decrease was observed in CZ4979X vs CZ4810X (*p*-value = 0.08, ME = -0.73) (Fig. 7A, B). Expectedly, consistent directional changes were present for theoretical yield, with biostimulant demonstrating a marginal increase (*p*-value = 0.09, ME = 464.66) over the control, and CZ4979X being decreased in comparison to CZ4979X (albeit insignificantly) (Fig. 7A, B). Above- and belowground biomass showed opposing trends between cultivars, being statistically increased (*p*-value = 0.002, ME = 3.66) and insignificantly decreased, respectively, in CZ4979X vs CZ4810X (Fig. 7A, B). Both parameters were increased insignificantly in biostimulant vs control (Fig. 7A, B). Lastly, pods/plant was increased significantly in CZ4979X vs CZ4810X (*p*-value = 0.02, ME = 14.23) and increased insignificantly in biostimulant vs control (Fig. 7A, B). The cultivar explained the highest proportion of variance for aboveground biomass and pods/plant (marginalized R^2^ = 0.85 and 0.71, respectively), while variance in 100-seed weight, belowground biomass, and theoretical yield were best explained by treatment (marginalized R^2^ = 0.56, 1.0, and 0.95, respectively).

Phenotype-taxon networks were constructed and visualized as described for edaphic parameters, encompassing 128 ± 6.66 nodes (eukaryotic *n* = 75.20 ± 5.64, prokaryotic *n* = 52.80 ± 1.56) and 448.20 ± 35.11 edges (Fig. 7C–E). Furthermore, 148 nodes (89 eukaryotes, 59 prokaryotes) were present in one or more of the networks (Fig. 7C, D). Consistent with the previous analysis, node prioritization identified 17 eukaryotic genera that are predominantly aquatic/partly aquatic with mycelial growth and a saprotrophic lifestyle, seven of which are also annotated as plant pathogens (Fig. 7F, G). The remaining genera included the prokaryotes *Bradyrhizobium*, *Burkholderia*, and *Lysobacter* (Fig. 7F, G). Six of the 20 were represented in the core microbiome. In addition, 10 (50%) nodes were prioritized for both agronomic and edaphic networks. Rank-based measures of association were next inferred between the top 20 nodes and between nodes and agronomic parameters. There were 35 significant associations between nodes (positive *n* = 31, negative *n* = 4), and *Neocosmospora* was most represented (positive *n* = 8, negative *n* = 1, total *n* = 9) (Fig. 7F). Additionally, six significant associations were discerned between nodes and parameters, with four nodes positively associated with aboveground biomass (*Naegleria*, *Setophoma*, *Neonectria*, and *Fusariella*), *Leptosphaeria* positively associated with pods/plant, and *Phaeosphaeriopsis* associated negatively with theoretical yield (Fig. 7G).

## Discussion

Soil is the most biodiverse habitat on Earth, harboring an estimated 59% of all living organisms [130]. Yet, relatively little is known about the inhabitants of this dynamic ecosystem, their interaction, their collective influence on environmental (and thereby human) health, and the interplay of stochastic and deterministic processes shaping such communities [131–133]. Single-molecule-based sequencing stands at the forefront of technologies predicted to clarify these ambiguities inherent in complex microbial systems [134]. To this end, the current study paired the commercial LoopSeq SLR platform with avidity sequencing to profile both eukaryotic and prokaryotic fractions of the soybean rhizosphere microbiome. An in situ experimental design reflected potential environmental dependencies in microbiome structure, which are likely missed in greenhouse/growth chamber experiments [135, 136], yet remain indispensable for practical application of derived inferences [137]. Multiple growth stages, commercial cultivars (genotypes), and biostimulants were also incorporated given their reported effect on rhizosphere microbiome assembly in soybean [13, 17, 18] and other plant systems [138–140]. The aim of this approach was to generate a well-resolved depiction of soybean rhizosphere microbiome structure and composition, laying groundwork for future applications in microbiome-based agriculture.

Perhaps the most significant outcome of this study was the taxonomic resolution achieved with both 16S and 18S-ITS SLRs. Traditional short-read amplicon sequencing rarely classifies ASVs beyond genus level, constraining biological inference [29]. For instance, Sugiyama et al. [141] suggested that soybean demonstrates species- and even strain-level selection of *Bradyrhizobia* based upon stark abundance patterns of ASVs/OTUs with *Bradyrhizobium* annotation; yet, this notion could not be verified given the limited resolution discerned with pyrosequencing. In the present work, assembling all 9 hypervariable regions of the 16S rRNA gene assigned prokaryotes to at least species level, with nearly one-fourth of mapped, full-length ASVs obtaining strain-level classification. This included the identification of 13 *Bradyrhizobium* species, some of which demonstrated exclusivity and/or differential abundance (i.e., putative selection) across experimental conditions. Furthermore, a subset of ASVs corresponding to *Bradyrhizobium elkanii* and *Bradyrhizobium japonicum* (genus members with the greatest absolute abundance) were resolved at strain level (one and four strains, respectively). This result further coincides with prior studies wherein *Bradyrhizobium elkanii* and *Bradyrhizobium japonicum* were the predominant species to nodulate soybean [142].

In eukaryotic microbial community analysis, the de novo-assembled 18S-ITS1-ITS2 molecules facilitated genus-level taxonomic assignment for all mapped ASVs, and species level assignment for approximately 45%. This strategy effectively captured diverse fungal taxa with agricultural importance, such as soybean-parasitizing genera. Beyond fungi, the analysis identified five kingdoms encompassing 19 genera of protists, including *Phytophthora* and *Pythium* [143]. Assessing soil-dwelling prokaryotic, fungal, and protist communities in tandem bears significance given that general primers do not exist for short-read amplicon profiling of protists [19, 144] and the understated yet significant role of protists in the soil microbiome [145]. Furthermore, the resolution achieved here permitted the automated retrieval of taxonomy-based functional annotations, allowing for highly reproducible biological inference without the need for sequence-based functional prediction.

Measures of α and β diversity suggested an overarching temporal effect on microbiome structure and composition, with more subtle trends attributed to treatment, cultivar, and fixed effect interaction. These findings were consistent with Moroenyane et al. [146], wherein spatial and temporal dynamics were key modulators of α and β diversity in the soybean rhizosphere microbiome. Further, α diversity aligned explicitly with the work of Longley et al. [13]. In both studies, eukaryotic and prokaryotic richness were decreased at the R2 growth stage and increased by R6 [13]. Shannon diversity trends matched the no-till soil findings of Longley et al. [13], showing reduced eukaryotic diversity at R6 compared to R2, with prokaryotes exhibiting the inverse trend. The authors of the compared study noted that their results deviated from prior research, postulating that management could account for the discrepancy [13]. In this regard, the accordance between the current and prior work may be attributed to the absence of tillage in both experimental designs. Agreeance may also reflect growth stage selection, as bacterial diversity in the soybean rhizosphere has been evidenced to increase between R1 and R5 and then decrease from R5 to R8 [147]. Moreover, the sole use of full-length contigs for analysis may have impacted diversity estimates, potentially excluding shorter sequences that contribute to overall α and β diversity.

Consistent with previous studies, the eukaryotic microbiome composition was predominantly Ascomycota [9, 13], while the prokaryotic fraction was largely represented by Proteobacteria [9, 18], as evidenced by taxonomic classification of mapped ASVs. The β diversity patterns echoed findings from Moroenyane et al. [146] in which growth stage (and interactions comprised thereof) best explained compositional dissimilarity, yet also displayed significant heterogeneous dispersion, across both eukaryotic and prokaryotic communities. In the current study, a spatial effect was evident in the eukaryotic microbiome composition, with samples clustering by field location. Given the experimental setup replicated a conventional row crop system, this may reflect an "edge-of-field" effect, with plots near turnrows receiving varied moisture or amendment applications. Comparable findings were reported by Longley et al. [13] wherein management strategy (i.e., conventional, no-till, organic) rendered distinct clustering of eukaryotic rhizosphere communities, with such trends absent for prokaryotic communities [13]. Notably, heterogeneity arising from field location was controlled statistically in all analyses.

Community membership revealed core, unique, and differentially abundant taxa across fixed effect levels. The core microbiome is a crucial element for rhizosphere microbiome assembly and consequent plant growth promotion [148, 149]. Thus, it is unsurprising that the core microbiome in this study was enriched with saprotrophic fungi, which decompose organic matter, contribute to nutrient cycling, and support soil structure [150]. Unique taxon identification reinforced the supposition of Sugiyama et al. [141] that *Bradyrhizobia* are subject to species-level selection, and implicated strong host selectivity of parasites/pathogens and mutualists. With regard to the latter, numerous plant pathogens were exclusive to the rhizosphere of the SDS-susceptible soybean cultivar, particularly at later growth stages. This could be attributed to compromised defense mechanisms of the susceptible cultivar, allowing opportunistic pathogens to colonize and proliferate, or possibly due to specific root exudates from this cultivar that inadvertently promote the growth of these pathogens. The exclusivity of *Streptomyces panaciradicis*, *Pseudomonas fluorescens*, and *Streptomyces griseoplanus* in the SDS-tolerant rhizosphere may also reflect host selection, as the two former have been leveraged as biocontrol agents against *Fusarium* pathogens [151, 152] and the latter as a biocontrol agent against *Macrophomina* [153]. The *Pseudomonas* genus has also been associated with SDS-suppressive soils spanning 45 soybean fields [10]. Lastly, the enrichment of *Bradyrhizobia* in CZ4979X vs CZ4810X and in Biostimulant vs Control further supports the exclusivity of *Pseudomonas fluorescens*, which is well-evidenced to interact synergistically with *Bradyrhizobium japonicum* [154, 155]. These microbial dynamics in the soybean rhizosphere highlight potential avenues for targeted crop protection, improved soil health, and optimized disease-resistant breeding.

Putative co-occurrences between prokaryotic, fungal, and protist genera were determined using Spearman and Pearson association methods. The Pearson networks exhibited greater complexity and more pronounced variability in node relative abundance compared to the Spearman networks. This disparity could be influenced by Spearman's method of assigning similar rank values to taxa with minimal or zero abundances, leading to simpler network structure and a reduced representation of high-abundance taxa [156]. Conversely, Pearson's sensitivity to actual data magnitudes may amplify the presence of notably abundant taxa, resulting in networks with a broader range of densities and node abundances [156]. Nonetheless, the dominant effect of treatment-cultivar interaction on condition-specific network structure was persistent across the association methods. In like manner, Liu et al. [18] noted a subtle genotype effect on soybean rhizosphere microbial co-occurrence network structure. Due to the reported effects of biostimulant application on soybean agronomic performance [157] and microbial network structure in other environments [158], it is also logical to presume its influence on network structure in the present work. Still, one must consider such findings as preliminary, given the shortcomings in inferring ecological interaction from co-occurrence [159] and that mapped ASVs were used exclusively for co-occurrence network construction, the latter of which could influence network structure and node prioritization. It is therefore recommended to complement network analysis with additional measurements for more robust hypothesis-driven research [160].

In this manner, 24 edaphic measurements were collected for each soil sample, evaluated with GLMMs and rank-based associations, and incorporated into phenotype-taxon networks. Soil organic matter (SOM) was among the most dynamic parameters assessed, displaying significant variation between five fixed effect level comparisons. This may reflect robust organic macromolecule depolymerization given the observed enrichment of saprotrophs in the eukaryotic rhizosphere microbiome [161] and Proteobacteria in the prokaryotic fraction [162], and perhaps coincides with the establishment of nodulation [163]. The edaphic data were used independently to construct phenotype-taxon networks based on the framework of Poudel et al. [89]. A more exhaustive approach was used to prioritize nodes by modularity and centrality, accentuating microbial taxa with both module-specific and network-wide influence. As expected, the prioritized nodes for edaphic networks were mostly saprotrophic eukaryotes. Moreover, the core rhizosphere microbiome has been shown to interact with more transient taxa via competition and cooperation, being central for microbial network structure and functional stability [149, 164]. In line with this, nearly one-third of the prioritized nodes for edaphic networks were members of the core microbiome. Other identified nodes reinforced trends in edaphic measures (e.g., the β-Proteobacteria genus *Burkholderia* and the α-Proteobacteria genus *Bradyrhizobium* are prominent lignin decomposers that can nodulate soybean [162, 165]).

The microbiome dataset was further contextualized by taking agronomic measurements at the end of the growing season. The most apparent trend was that biostimulant application increased every measured trait, with variation in 100-seed weight and theoretical yield being statistically significant. Additionally, the SDS-susceptible variety had heightened 100-seed weight and theoretical yield in comparison to the tolerant cultivar despite having reduced pods/plant, aboveground biomass, and increased pathogens in the rhizosphere, implicating a putative fitness cost associated with genetic resistance/tolerance in the absence of disease [166]. Notably, each replicate for 100-seed weight and theoretical yield encompassed approximately 40 plants in a manner aligned with yield plot trials. Network analysis and node prioritization were consistent with that for edaphic properties, highlighting saprotrophic eukaryotes, SOM-decomposing/nitrogen-fixing bacteria, and members of the core microbiome. As evidenced, complementing co-occurrence networks with phenotypic data provides improved ecological context that can guide the practical application of derived inferences (e.g., through the design and implementation of synthetic microbial communities) [89].

## Conclusions

The defined study provides a taxonomically resolved view of the soybean rhizosphere microbiome. Unique in its design, this research was carried out in situ, circumventing the often-observed discrepancy where taxa linked to host fitness in controlled settings fail to replicate symbiont status under field conditions [136]. This study revealed that both eukaryotic and prokaryotic rhizosphere microbiomes display structural and compositional variation in response to treatment, cultivar, and growth stage, consistent with earlier studies primarily leveraging short-read sequencing. Furthermore, the novelty of the present work was well-accentuated through community membership analysis, where taxonomic resolution permitted taxonomy-based functional annotation, identifying an ecologically relevant, saprotroph-rich core microbiome and demonstrating empirical evidence for host selection of mutualistic taxa and concomitant pathogen restriction. The use of multiple association methods for microbial co-occurrence network construction and the comprehensive assessment of network topology underscored the influence of experimental conditions (biostimulant application and cultivar) on co-occurrence network structure. Moreover, the augmentation of such networks with edaphic and agronomic data, complemented by regularized linear regression and a novel node prioritization criterion, identified microbial genera which may be leveraged for sustainable agriculture, many of which are known for their ecological significance. In conclusion, the application of synthetic long-read technology and an in situ experimental design yielded an unparalleled understanding of the soybean rhizosphere microbiome, signifying a considerable advancement in crop microbiome research with practical implications for microbiome-based agriculture.

### Supplementary Information

Additional file1 

Additional file2 

Additional file3 

Additional file4 

Additional file5 

Additional file6 

Additional file7 

## Data Availability

The 16S and 18S-ITS FASTQ files are available in the NCBI Sequence Read Archive database (16S BioProject ID: PRJNA1062839; 18S-ITS BioProject ID: PRJNA1063224). R Markdown and RData files required for the reproduction of this work are available at https://github.com/brett-hale/Hale_2024_SLR.git.
