# Supplementary material for: Fine-scale characterization of the soybean rhizosphere microbiome via synthetic long reads and avidity sequencing
Source: Environ Microbiome. 2024 Jul 12;19:46. doi: 10.1186/s40793-024-00590-5 (PMC11241880; doi:10.1186/s40793-024-00590-5)
Supplement: Supplementary file 2 — Additional file2 [file 40793_2024_590_MOESM2_ESM.pdf]

*Single-molecule-based characterization of the soybean rhizosphere microbiome

Brett Hale[1,2,3], Caitlin Watts[4, #], Matt Conatser[4], Edward Brown[4], and Asela J. Wijeratne[2,3,*]

[1] AgriGro Incorporated, Doniphan, MO, USA

[2] Arkansas Biosciences Institute, Arkansas State University, State University, AR, USA

[3] College of Science and Mathematics, Arkansas State University, State University, AR, USA

[4] College of Agriculture, Arkansas State University, State University, AR, USA

[#] Current address: Department of Animal Sciences, Purdue University, West Lafayette, IN, USA

[*] Correspondence: awijeratne@astate.edu

*Title changed to "Fine-scale characterization of the soybean rhizosphere microbiome via synthetic long reads and avidity sequencing" per reviewer request.

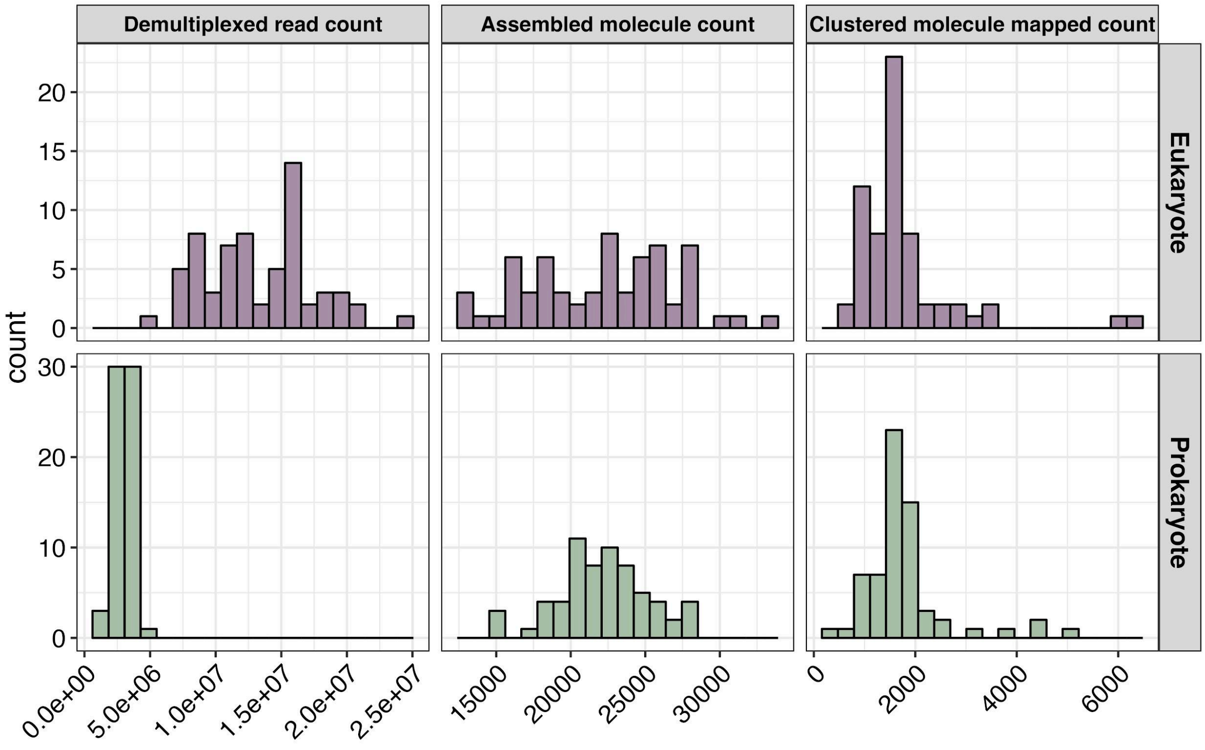

Supplementary Figure 1 SLR assembly and mapping statistics for 18S-ITS (purple, "Eukaryote") and 16S (green, "Prokaryote") datasets. **Left** demultiplexed short read count. **Middle** Assembled molecule (SLR) count. **Right** Clustered molecule mapped count (number of mapped SLRs per sample).

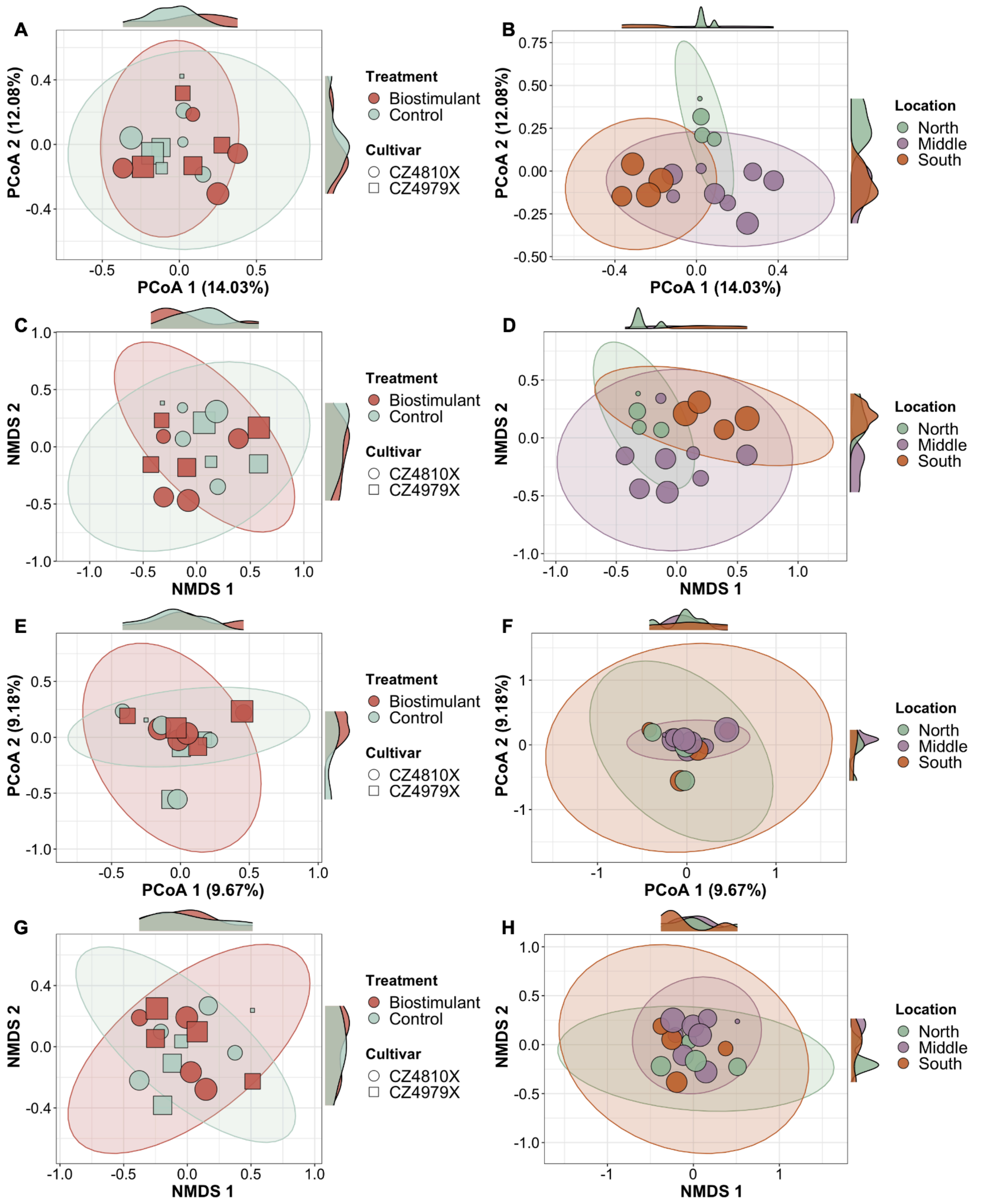

Supplementary Figure 2 PCoA and NMDS ordinations of Bray-Curtis dissimilarity for the baseline (V1 growth stage). **A**, **B** PCoA ordinations of eukaryotic dissimilarity. **C**, **D** NMDS ordinations of eukaryotic dissimilarity. **E**, **F** PCoA ordinations of prokaryotic dissimilarity. **G**,

H NMDS ordinations of prokaryotic dissimilarity. Point size reflects Shannon diversity index.

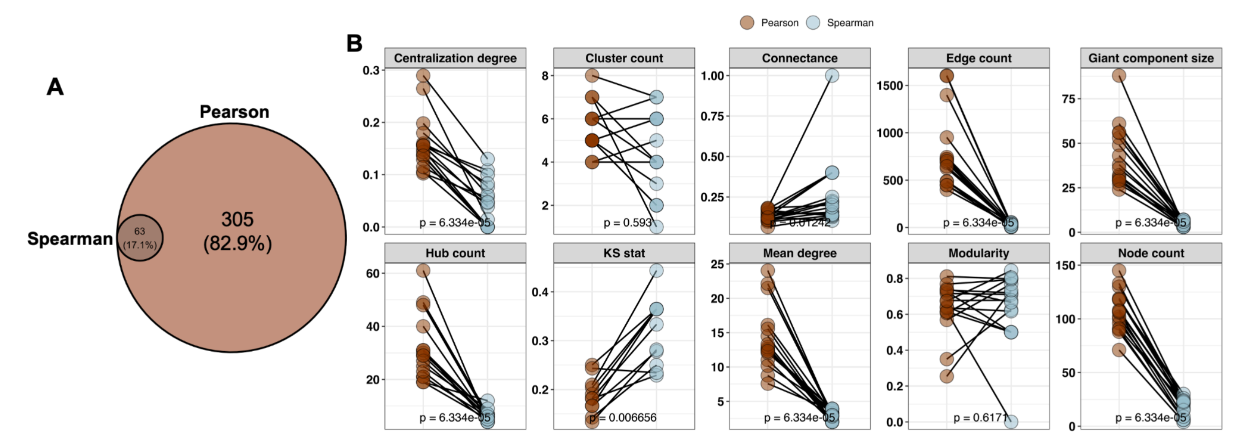

Supplementary Figure 3 A comparison of condition-specific co-occurrence networks constructed with Spearman and Pearson associations. **A** Node (genus) representation across Spearman and Pearson networks. **B** Comparison of network topologies between the association methods, with each point representing a condition-specific network. *P*-values were obtained from Friedman rank sum tests and used to assess the differential ranking of conditions between association methods for each topological feature.

Supplementary Table 1 Eukaryotic β diversity succeeding biostimulant application									
		**PERMANOVA**				**Dispersion**			
**Distance matrix**	**Variable**	**Df**	**R2**	**F**	***p*-value**	**Df**	**Mean Sq**	**F**	***p*-value**
Bray-Curtis	Treatment	1	0.02253	1.0812	0.1183	1	0.0006474	0.0824	0.7754
	Cultivar	1	0.02416	1.1596	0.0639	1	0.0049387	0.6314	0.4309
	Growth Stage	2	0.08332	1.9993	**0.0001**	2	0.033854	6.3216	**0.003806**
	Treatment:Cultivar	1	0.01576	0.7565	0.829	3	0.0017469	0.2109	0.8883
	Treatment:Growth Stage	2	0.03106	0.7452	0.9372	5	0.0139236	2.2702	0.0648
	Cultivar:Growth Stage	2	0.03137	0.7527	0.9223	5	0.0151566	2.6737	0.03471
Jaccard	Treatment	1	0.02207	1.0497	0.1271	1	0.0002231	0.0612	0.8057
	Cultivar	1	0.02349	1.1169	**0.0482**	1	0.0026749	0.7362	0.3953
	Growth Stage	2	0.06705	1.5945	**0.0001**	2	0.0155302	5.9657	**0.005033**
	Treatment:Cultivar	1	0.01742	0.8285	0.888	3	0.0008659	0.2226	0.8802
	Treatment:Growth Stage	2	0.0357	0.849	0.9113	5	0.0065309	2.1587	0.077
	Cultivar:Growth Stage	2	0.03525	0.8383	0.9404	5	0.0071203	2.5899	**0.03951**
Euclidean	Treatment	1	0.02291	1.0966	0.1033	1	3127208	2.2306	0.1421
	Cultivar	1	0.01936	0.9267	0.5775	1	344921	0.2339	0.631
	Growth Stage	2	0.06131	1.4675	**0.0001**	2	1226935	0.8464	0.4357
	Treatment:Cultivar	1	0.02266	1.0848	0.1214	3	1194043	0.8263	0.4865
	Treatment:Growth Stage	2	0.03999	0.9572	0.4702	5	1792230	1.3179	0.2751
	Cultivar:Growth Stage	2	0.03989	0.9547	0.4977	5	1861665	1.3522	0.2616
P-values ≤ 0.05 are bolded.									

**Supplementary Table 2 Prokaryotic β diversity succeeding biostimulant application**									
		**PERMANOVA**				**Dispersion**			
**Distance matrix**	**Variable**	**Df**	**R2**	**F**	***p*-value**	**Df**	**Mean Sq**	**F**	***p*-value**
Bray-Curtis	Treatment	1	0.0116	0.5284	0.9899	1	0.00008423	0.0806	0.7778
	Cultivar	1	0.01827	0.8326	0.7134	1	0.00001609	0.0121	0.9131
	Growth Stage	2	0.02712	0.6179	0.9919	2	0.0023488	2.0362	0.1424
	Treatment:Cultivar	1	0.01262	0.5748	0.974	3	0.00073918	0.5185	0.6718
	Treatment:Growth Stage	2	0.05735	1.3064	0.075	5	0.00090551	0.3373	0.8874
	Cultivar:Growth Stage	2	0.03896	0.8876	0.6969	5	0.0015852	0.7743	0.5738
Jaccard	Treatment	1	0.01437	0.6608	0.9923	1	0.00000011	3.00E-04	0.986
	Cultivar	1	0.0183	0.8412	0.7969	1	0.00000078	0.0016	0.9682
	Growth Stage	2	0.03153	0.7246	0.995	2	0.00061552	1.5084	0.2322
	Treatment:Cultivar	1	0.0146	0.6712	0.9886	3	0.00017742	0.382	0.7665
	Treatment:Growth Stage	2	0.05418	1.2454	0.0521	5	0.00030544	0.2352	0.9448
	Cultivar:Growth Stage	2	0.04036	0.9276	0.7052	5	0.00044362	0.5736	0.7198
Euclidean	Treatment	1	0.0083	0.3766	0.9667	1	954334	0.5029	0.4818
	Cultivar	1	0.01736	0.7877	0.6436	1	1612258	0.8619	0.358
	Growth Stage	2	0.0229	0.5193	0.9683	2	705407	0.367	0.6948
	Treatment:Cultivar	1	0.00608	0.2757	0.9922	3	1340019	0.7047	0.5543
	Treatment:Growth Stage	2	0.06798	1.5418	0.0955	5	705047	0.3792	0.8601
	Cultivar:Growth Stage	2	0.03972	0.9008	0.5992	5	1194020	0.6364	0.6731

Supplementary Table 3 β diversity preceding biostimulant application									
**Eukaryote**									
		**PERMANOVA**				**Dispersion**			
**Distance matrix**	**Variable**	**Df**	**R2**	**F**	***p*-value**	**Df**	**Mean Sq**	**F**	***p*-value**
Bray-Curtis	Treatment	1	0.07415	1.0967	0.0745	1	0.0017845	1.0289	0.3276
	Cultivar	1	0.05798	0.8576	0.5268	1	0.0018172	0.7677	0.3957
	Treatment:Cultivar	1	0.05653	0.8361	0.5806	3	0.0012842	0.3876	0.764
Jaccard	Treatment	1	0.07044	1.047	0.0771	1	0.0006489	0.9681	0.3419
	Cultivar	1	0.06124	0.9104	0.5567	1	0.00072719	0.8262	0.3788
	Treatment:Cultivar	1	0.06104	0.9074	0.559	3	0.00050409	0.3913	0.7614
Euclidean	Treatment	1	0.06784	1.0199	0.2167	1	153509	3.3588	0.0882
	Cultivar	1	0.06987	1.0504	0.1737	1	3310	0.0626	0.8061
	Treatment:Cultivar	1	0.0641	0.9637	0.3355	3	51245	1.2509	0.3348
**Prokaryote**									
Bray-Curtis	Treatment	1	0.07804	1.1107	0.3665	1	0.00015172	0.0561	0.8162
	Cultivar	1	0.03857	0.549	0.9642	1	0.00005489	0.0503	0.8258
	Treatment:Cultivar	1	0.04029	0.5735	0.9576	3	0.0002761	0.289	0.8325
Jaccard	Treatment	1	0.07697	1.1153	0.3151	1	0.00005263	0.0314	0.8619
	Cultivar	1	0.04694	0.6802	0.961	1	0.00000607	0.0162	0.9006
	Treatment:Cultivar	1	0.04792	0.6943	0.953	3	7.39E-05	0.2854	0.835
Euclidean	Treatment	1	0.08829	1.2677	0.4179	1	17143502	4.4814	0.05266
	Cultivar	1	0.04717	0.6772	0.696	1	2186234	0.4266	0.5242
	Treatment:Cultivar	1	0.02876	0.4129	0.9525	3	7371224	1.908	0.1822
P-values ≤ 0.05 are bolded.									

**Supplementary Table 4 β diversity succeeding biostimulant application (all full-length ASVs)**									
**Eukaryote**									
		**PERMANOVA**				**Dispersion**			
**Distance matrix**	**Variable**	**Df**	**R2**	**F**	***p*-value**	**Df**	**Mean Sq**	**F**	***p*-value**
Bray-Curtis	Treatment	1	0.02360	1.1032	**0.0172**	1	0.0003830	0.2269	0.6361
	Cultivar	1	0.02213	1.0347	0.0658	1	0.0012835	0.888	0.3509
	Growth Stage	1	0.04697	1.0981	**0.0027**	2	0.0029409	2.4037	0.1019
	Treatment:Cultivar	1	0.01864	0.8714	0.7651	3	0.0005058	0.2768	0.8418
	Treatment:Growth Stage	2	0.03810	0.8906	0.7306	5	0.0013215	0.728	0.6063
	Cultivar:Growth Stage	2	0.03776	0.8827	0.7983	5	0.0018223	1.5647	0.191
**Prokaryote**									
Bray-Curtis	Treatment	1	0.01733	0.8118	0.8063	1	0.0002981	0.1765	0.6764
	Cultivar	1	0.02196	1.0289	0.2702	1	0.0002841	0.1671	0.6846
	Growth Stage	2	0.05064	1.1860	**0.0246**	2	0.0062601	3.254	**0.04788**
	Treatment:Cultivar	1	0.01509	0.7067	0.9686	3	0.0004848	0.2731	0.8445
	Treatment:Growth Stage	2	0.04747	1.1120	0.0784	5	0.0023105	0.8585	0.5168
	Cultivar:Growth Stage	2	0.03635	0.8516	0.8136	5	0.0031967	1.3312	0.2698
P-values ≤ 0.05 are bolded.									

**Supplementary Table 5 β diversity preceding biostimulant application (all full-length ASVs)**									
**Eukaryote**									
		**PERMANOVA**				**Dispersion**			
**Distance matrix**	**Variable**	**Df**	**R2**	**F**	***p*-value**	**Df**	**Mean Sq**	**F**	***p*-value**
Bray-Curtis	Treatment	1	0.06111	0.8899	0.4059	1	0.0000085	0.0133	0.9097
	Cultivar	1	0.05928	0.8632	0.7089	1	0.0000474	0.0624	0.8063
	Treatment:Cultivar	1	0.05555	0.8089	0.9894	3	0.0000627	0.043	0.9875
**Prokaryote**									
Bray-Curtis	Treatment	1	0.07233	1.0366	0.2751	1	0.0000632	0.0521	0.8227
	Cultivar	1	0.04507	0.6460	0.9695	1	0.0000051	0.0055	0.9422
	Treatment:Cultivar	1	0.04530	0.6492	0.9669	3	0.0002416	0.2462	0.8625

P-values ≤ 0.05 are bolded.
