# Supplementary material for: Fine-scale characterization of the soybean rhizosphere microbiome via synthetic long reads and avidity sequencing
Source: Environ Microbiome. 2024 Jul 12;19:46. doi: 10.1186/s40793-024-00590-5 (PMC11241880; doi:10.1186/s40793-024-00590-5)
Supplement: Supplementary file 7 — Additional file7 [file 40793_2024_590_MOESM7_ESM.docx]

**Additional Files**

**Additional File 1**

- File format- Excel file (.xls)
- Title of data- Additional File 1
- Description of data- Weather data for the growing season, as well as region-specific historical weather data.

**Additional File 2**

- File format- PDF (.pdf)
- Title of data- Additional File 2
- Description of data
  - **Supplementary Figure 1** SLR assembly and mapping statistics for 18S-ITS and 16S datasets.
  - **Supplementary Figure 2** PCoA and NMDS ordinations of Bray-Curtis dissimilarity for the baseline (V1 growth stage).
  - **Supplementary Figure 3** A comparison of condition-specific co-occurrence networks constructed with Spearman and Pearson associations.
  - **Supplementary Table 1** Eukaryotic β diversity succeeding biostimulant application.
  - **Supplementary Table 2** Prokaryotic β diversity succeeding biostimulant application.
  - **Supplementary Table 3** β diversity preceding biostimulant application.
  - **Supplementary Table 4** β diversity succeeding biostimulant application (all full-length ASVs).
  - **Supplementary Table 5** β diversity preceding biostimulant application (all full-length ASVs).

**Additional File 3**

- File format- Excel file (.xls)
- Title of data- Additional File 3
- Description of data- SLR assembly and mapping statistics for 18S-ITS data.

**Additional File 4**

- File format- Excel file (.xls)
- Title of data- Additional File 4
- Description of data- SLR assembly and mapping statistics for 16S data.

**Additional File 5**

- File format- Excel file (.xls)
- Title of data- Additional File 5
- Description of data- Unique taxa across fixed effect levels.

**Additional File 6**

- File format- Excel file (.xls)
- Title of data- Additional File 6
- Description of data- Differentially abundant taxa across fixed effect levels.
